# CurmElo: The theory and practice of a forced-choice approach to producing preference rankings

**DOI:** 10.1371/journal.pone.0252145

**Published:** 2021-05-27

**Authors:** Soham Sankaran, Jacob Derechin, Nicholas A. Christakis

**Affiliations:** 1 Yale Institute for Network Science, Yale University, New Haven, Connecticut, United States of America; 2 Sociology, Yale University, New Haven, Connecticut, United States of America; 3 Computer Science, Cornell University, Ithaca, New York, United States of America; 4 Pashi, San Francisco, California, United States of America; UCLA Fielding School of Public Health, UNITED STATES

## Abstract

We introduce CurmElo, a forced-choice approach to producing a preference ranking of an arbitrary set of objects that combines the Elo algorithm with novel techniques for detecting and correcting for (1) preference heterogeneity induced polarization in preferences among raters, and (2) intransitivity in preference rankings. We detail the application of CurmElo to the problem of generating approximately preference-neutral identifiers, in this case four-letter and five-letter nonsense words patterned on the phonological conventions of the English language, using a population of Amazon Mechanical Turk workers. We find evidence that human raters have significant non-uniform preferences over these nonsense words, and we detail the consequences of this finding for social science work that utilizes identifiers without accounting for the bias this can induce. In addition, we describe how CurmElo can be used to produce rankings of arbitrary features or dimensions of preference of a set of objects relative to a population of raters.

## Introduction

In this paper, we detail the theory and practice of CurmElo, a forced-choice based approach to producing a preference ranking of an arbitrary set of objects. CurmElo was originally designed for the purpose of producing sets of approximately preference-indifferent identifiers, which we define as identifiers that are relatively equally preferred across a population of subjects. In our original use case, those identifiers were sets of nonsense words of four and five letters.

This work has three motivations. The first motivation is that when eliciting preference, forced-choice based questions are preferable to Likert-style scales in a number of circumstances. The second motivation is that confounding preference for identifiers of various kinds rears its head in numerous unexpected places in social science research, and that it is essential to use some explicit form of preference elicitation, ideally using the population targeted by the research as raters, to control for these effects. The third is that preference heterogeneity induced polarization in preferences among raters and also intransitivity in preference rankings can render naive attempts to control for identifier preference inadequate, and that some method for dealing with these issues is necessary before the preference rankings can be used.

In the section below, we outline the three topics and detail our initial motivating use case for CurmElo, the production of approximately preference-indifferent four-letter and five-letter nonsense identifiers. In the rest of the paper, we use this motivating use case to demonstrate how CurmElo incorporates these insights into a comprehensive method for preference elicitation.

### Motivations

#### Why forced choice?

We are looking to elicit preference data on a large number of unknown identifiers in an environment where we are concerned about bias. When eliciting preferences from users it is common to use either Likert-type scales [1] or Forced Choice Paired Comparisons. Each method has its own unique strengths and weaknesses and which method is preferable depends on the context. In psychometric contexts direct comparisons between Forced Choice and Likert Scales found Forced Choice was found to be less biased that Likert Scales, but it was also found to be less reliable, suggesting that larger sample sizes may be necessary for Forced Choice measures. [2–4] One a different comparison of Likert Scales and Forced Choice in the context of measuring achievement motivation found the Likert measures out preformed forced choice in terms of validity. [5] Evidence suggests that raters do not always consider both alternatives and that results from forced choice comparisons contain both relative and absolute data, suggesting the potential for bias in Forced Choice settings. [6] Alternatively other research suggests that Forced Choice does a better job at predicting real world outcomes than Likert Scales [7, 8] and this real world impact has been noted publicly described but unpublished work within the technology industry [9].

When using Likert scales, the choice of scale can impact the outcomes. It has been found that the effect of question wording (positive vs. negative wording) does not generalize across different scales, which can make it difficult to compare results between measures that use different scales. [10] As a result, there is some controversy around the use of such scales, especially single Likert questions as opposed to comparisons across multiple questions, to measure preference and sentiment [11–13]. Additionally, within the Likert scale literature, there are significant inconsistencies about what the optimal size of the scale is. Some empirical results suggest that, consistent with the predictions of information theory, scales with greater numbers of points(1–7 vs 1–11 for example) are better [14], while other empirical results suggest precisely the opposite, that scales with more points tended to be less reliable. [15, 16] Moreover, the optimal parity—even or odd—of the scale used is also contentious: while a sizeable number of deployments of Likert-type surveys appear to use odd-parity scales, research into these instruments suggests that survey participants will often times use a middle option, only available on an odd-parity scale, to express that they don’t know or don’t have an opinion about the question instead of an actual opinion corresponding with the middle value, even when an “I don’t know” option is available, in many cases materially changing the final results [17]. Forced choice surveys can run into the opposite problem, by not providing an “I don’t know” or no preference option it is difficult to differentiate between indifference and identically [18].

In our particular use case, a Likert-based preference elicitation method would likely be even more unreliable due to the unfamiliarity of the raters with the identifiers they are being asked to compare—unlike familiar objects like actual English words or, say, human faces, they may have no solid internal baseline for preference for these nonsense words, whereas comparing two identifiers requires no such preexisting knowledge or baseline. Given that reducing bias is imperative in our setting, and that empirically Forced Choice methods have preformed well in settings similar to ours (with large numbers of unknown identifiers) [9], we used Forced Choice methods for Curmelo because we expect to to preform best in this setting.

#### Why preference-rank identifiers?

CurmElo was originally designed for the purpose of producing sets of approximately preference-indifferent identifiers, which we define as identifiers that are relatively equally preferred across a population of subjects. In our original use case, those identifiers were sets of nonsense words of four and five letters. While at first glance it may seem reasonable to expect that preference across a set of nonsense words generated randomly will not differ significantly, it is well established that people have innate preferences for particular numbers, letters, and strings of numbers and letters—examples of this include the name-letter effect, where people prefer letters in their own name over others [19], and the people’s documented preference for the number seven over other single-digit numbers [20, 21]. Research from cognitive science suggests that the map between the form of a word and its meaning is not entirely arbitrary [22], and that human raters impute category information to nonsense words in systematically different ways [23]. The existence of these preferences is also illustrated by work on the passwords people choose for online services [24, 25].

It seems likely that this sort of identifier preference extends not just to nonsense words, but potentially to any class of object that might be used as an identifier: images, sounds, physical objects, colours, etc. There is work in psychology that suggests that novel and nonsense stimuli of many kinds can prime people just as much as sensical and familiar stimuli [26]. This has serious implications for the use of identifiers in experimental social science.

Here is non-exhaustive set of examples of experimental social science work where we believe that identifier preference may be a confounder: work employing the Minimal Group Paradigm [27–29], and more generally any work where groups need identifiers; work involving inter-subject interaction where subjects have identifiers; work involving goals or target that need identifiers (our motivating use case fits in this and the previous category); work involving participants reading or listening to narratives where identifiers are used for specific characters. In work of these kinds, we believe that identifier preference, if left unaccounted for, might significantly skew results by heterogeneously changing effect size on a per-identifier basis, as well as make replication difficult due to cross-population preference heterogeneity. We suspect that identifier preference may be unacknowledged confounder for a large number of experiments in these areas.

As such, we believe that preference needs to be explicitly dealt with in some fashion in any social science work where preference for identifiers can be a confounder. This may take several forms. In certain experiments, such as in our motivating use case, one might control for identifier preference by using approximately preference-indifferent identifiers. In other settings, it might be useful to produce identifiers that are quantifiably different, up to a specified tolerance, on some dimension of preference for the raters, for example to measure interaction effects between identifier preference and some other variable.

Preference-conscious identifier generation may also be of value in other empirical or applied circumstances where the objective is to name people, objects or places in such way as to accord them neutral or specific preference of some kind, such as in game design, fiction writing, and bias training.

In this paper, we detail the motivation for the development of CurmElo for our specific use case, that is, issues with identifier preference we observed in our network science experiments, as well demonstrate that randomness in identifier generation and selection do not sufficiently mitigate these effects. We then propose a workable solution.

#### Why consider preference heterogeneity induced polarization and intransitivity in preference rankings?

CurmElo uses a version of the Elo algorithm to convert a set of forced-choice binary comparisons within a set of objects into ratings for each of those objects to form a totally-ordered ranking of the set. Consider the case where we want to find preference-indifferent objects of some kind. If we were to interpret these rankings naively, we would extract a subset of objects from the middle of the ranking distribution that are sufficiently similar in rating and call those objects preference-indifferent. It may be the case, however, that some of these objects are not so much preference-indifferent as ‘polarizing’, that is, strongly preferred by one subset of the population and strongly dispreferred by another. This sort of heterogeneity in preference may be the result of some hard-to-detect form of population heterogeneity, and could be a significant confounder if the objects are being used as identifiers in experiments, for example.

CurmElo uses a novel technique based on counting breaks in win-percentage monotonicity in Elo rankings to detect latent heterogeneity and identify polarizing objects. Crucially, this method is distinct from other formulations of the latent population heterogeneity problem since we need to measure no identifying characteristics of the populations other than their choices [30], and as such this could be a valuable method of measuring population-level heterogeneity via preference.

Transitivity is the property that given, some objects *a*, *b*, and *c*, where *a* is preferred to and ranked above *b*, and *b* is ranked above and preferred to *c*, then a will be preferred to *c*. To see why breaks in transitivity matter, consider a case where we want to run an experiment to investigate the interaction between identifier preference and some other variable or test condition. Now imagine that our set of identifiers is objects *a*, *b*, and *c*, except that now there is a transitivity break manifesting as a preference cycle such that *c* is preferred to *a*. This would completely disrupt any attempt to use preference as an independent variable in the experiment since the ranking is no longer coherent—one cannot say, for example, that *a* is always most preferred since in this case this is dependent on what it is being compared to—and thus analysis of data collected using these identifiers can produce problematic results. CurmElo uses a technique based on counting breaks in preference transitivity in Elo rankings to identify sets of objects that break transitivity.

#### Our motivating use case: Four and five letter nonsense identifiers

As part of our ongoing work on human consensus, we needed to find arbitrary identifiers of four-letter and five-letter length to be used as subject and goal identifiers respectively in an experiment where subjects were communicating with each other about a set of goals. We initially decided to use a truncated version of each subject’s Amazon Mechanical Turk HIT ID, a somewhat arbitrary number-letter string, as subject identifiers, and to use integers from 1–12 to identify goals.

In our initial pilot experiments, we found strong preference effects for specific identifiers, in particular the numbers 9, 1, and 5, with multiple participants subjects clustering asymmetrically around specific identifiers. It has been found that when people are asked to pick a “random” number from an interval, there is clumping around specific parts of interval, so this result is consistent with what is already known [20, 21]. This made it very difficult isolate the mechanisms of the process we were investigating independently of the identifiers being used. Second, the random subject identifiers, despite their arbitrariness, seemed subject to heterogeneous preference effects that may have strongly influenced the likelihood of subjects to form consensus with other subjects based on their identifiers. These effects were both difficult to predict on a per-identifier basis and difficult to control for in our analysis.

Compounding these issues was the intelligibility of the subject identifiers—random number-letter strings such as ‘341AXM’ are not particularly easy to parse, pronounce, or remember. This effect made our experiment needlessly harder for participants, and may also have explained some of the heterogeneous preference effects given that subject identifiers that seemed closer to familiar words or names, such as ‘30EJON’ (not a real example), appeared to be more preferred.

Given this state of affairs, we set out to produce two distinct sets of identifiers that were both intelligible to our subjects and approximately preference-neutral across the population of subjects we were using for our experiment.

To produce our preference-indifferent identifiers, we first generated two very large sets of four and five-letter identifiers using formats based on the general rules of English phonology to ensure that they were reasonably pronounceable and memorable. For each set, we removed any identifier that had (as of January 2018) been used previously across the large and representative English-language Google Ngrams corpus [31]. Then, we used a version of the Elo rating system, initially formulated by Arpad Elo to rank chess players [32], to derive ratings for each identifier from individual pairwise comparisons to form a population-level ranking. After this, we applied a novel technique based on monotonicity breaks to remove identifiers that might be polarizing but achieve middling values in Elo ratings. Finally, we extracted a set of similarly-rated identifiers from the middle of the ranking distribution. These are our preference-indifferent identifiers.

Our approach allows us both to make claims about which identifiers are equally preferred by raters and also to make claims about which identifiers, overall, are more or less preferred by the raters. For instance, the following pairs of identifiers are equally preferred: (1) camaz and bumak; (2) lujaf and piqez; and (3) cixuq and quhuq. But the first pair is much liked by the raters, the third pair much disliked, and the middle pair has middling ratings (the subjects are neutral about neutral choices, as it were). Finally, we also provide (in the S4 Appendix) a list of 1,000 4-letter and 5-letter identifiers and their ratings, which might be useful to others, from social scientists to fiction writers, facing similar objectives.

## Materials and methods

This research was approved by the Yale Human Research Protection Program Institutional Review Boards IRB Protocol ID: 2000023887 This research contains no consent form as we did not collect personally identifying information.

### Preference data collection

We first formulated two sets of candidate identifiers using phonological formats that mimic name identifiers the English language [33].

For one set of identifiers, we generated all 4-character identifiers of the type:

vowel-consonant-vowel-consonant (VCVC).

For example: ayiz, erik.

There were 11025 of these four-letter identifiers in total.

For the second set of identifiers, we generated all 5-character identifiers of the type:

consonant-vowel-consonant-vowel-consonant (CVCVC).

For example: yezak, roman.

There were 231525 of these five-letter identifiers in total.

We then implemented the following procedure: (1) we removed any identifier that occurred once or more (as of January 2018) in the Google Ngram corpus of published work in English [31] (this left 118061 of the five-letter and 5969 of the four-letter identifiers), then (2) we randomly selected 1000 of the remaining identifiers from each of the sets.

**Fig 1:**
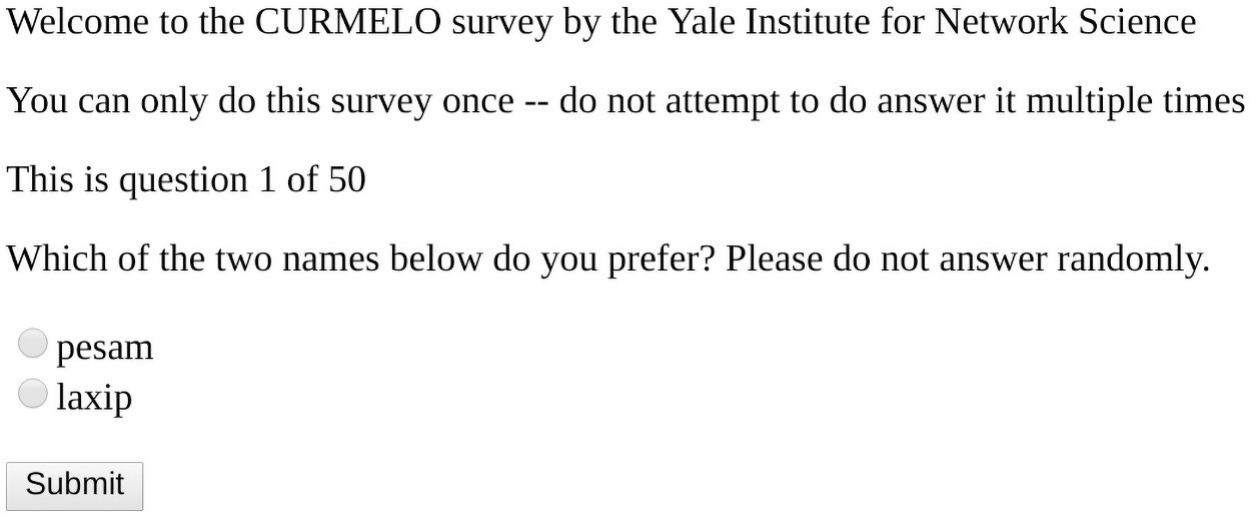
A screenshot of the CurmElo interface with two candidate CVCVC identifiers.

These sets of 1000 identifiers were then randomly matched up against each other (within and not across each set), in pairs on our custom-built CurmElo software platform. For each of the two sets, we used 400 unique US-based raters on Amazon Mechanical Turk to perform head-to-head preference comparisons of pairs of identifiers within the set. Note that these AMT raters were recruited from the same population from which we recruit participants in the experiments in which we would subsequently use the identifiers produced using this process, so we have high internal validity for the preference rankings. Each rater was shown 50 random pairs of non-identical identifiers, one pair at a time, and asked the following each time: “Which of the two names below do you prefer? Please do not answer randomly.” Fig 1 is a screenshot of the CurmElo interface.

There were 400 workers used for each set, and each worker was shown 50 pairs of identifiers. Given that there are 1000 identifiers in total, each identifier ended up with an average of 40 comparison data points. There is some variation in this number, but no identifier ended up with significantly fewer than 30 comparisons.

#### Querying other features and dimensions of preference using CurmElo

While in this use case, we asked raters which name identifier they preferred in general, one could use CurmElo to query any other specific feature or dimension of preference. For example, one might as “Which name sounds better?” or “Which name makes you happier?” or even “Which name seems reddest?”. If your objects are pictures of faces, one might ask “Which face appears angriest?” or “Which face is sharpest?”. The rankings created from the data thus collected would then correspond to the ranking of the objects relative to that specific feature (redness, anger, sharpness) or dimension of preference (sounds, happiness-of-feeling).

#### Using non-textual objects

CurmElo can be deployed for any set of objects that can practicably be exposed to raters. In an online-only setting such as Amazon Mechanical Turk, anything displayable on a webpage, including but not limited to audio, images, video, and interactive animations may be used. In a lab setting, physical objects may be used given that they can be uniquely identified and randomized systematically.

### Theory

#### The Elo algorithm

The Elo algorithm produces a relative rating across a set of objects. The algorithm is initialized by setting all objects to a some common initial rating, *R*_0_. Then, objects are matched against each other, with some external input determining an outcome where one objects ‘wins’ and the other ‘loses’. In CurmElo, a match is simply a comparison of two objects by a human participant (the external input) being asked to choose a winner and loser among them. Different applications may use different matching systems; for example, if Elo ratings are used for some sort of competitive activity, it may make sense to match objects—in this case players—with similar ratings. In our setting, we use random matching, as it allows the Elo ratings to quickly converge to their stationary distribution [34]. Consider objects *a* and *b* along with their corresponding Elo ratings R_a_ and R_b_. If *a* and *b* are matched, and object *a* wins, the ratings are updated as follows [32, 35]:

$$\begin{array}{c} R_{a}^{\prime }=R_{a}+k\frac{1}{1+e^{\frac{R_{a}-R_{b}}{R_{D}}}} \end{array}$$ (1)

$$\begin{array}{c} R_{b}^{\prime }=R_{b}-k\frac{1}{1+e^{\frac{R_{a}-R_{b}}{R_{D}}}} \end{array}$$ (2)

If *a* and *b* are matched, and object *b* wins, the ratings are updated as follows [32, 35]:

$$\begin{array}{c} R_{a}^{\prime }=R_{a}-k\frac{1}{1+e^{\frac{R_{b}-R_{a}}{R_{D}}}} \end{array}$$ (3)

$$\begin{array}{c} R_{b}^{\prime }=R_{b}+k\frac{1}{1+e^{\frac{R_{b}-R_{a}}{R_{D}}}} \end{array}$$ (4)

In this setting, k and R_D_ are free parameters, used to tune how sensitive that rating is to the results of new matches. It is possible to use a broader class of update functions other than $k\frac{1}{1+e^{\frac{R_{a}-R_{b}}{R_{D}}}}$ as long as it satisfies the conditions for a strong utility distribution, which will be discussed in the next section [35, 36]. We use the logistic update function because it is commonly used for Elo applications.

This process continues until all matches—in our case, comparisons—are complete, and we refer to the Elo ratings after all matches have occurred to be the “final Elo rating.” In contrast to applications in sports or gaming, where the number of matches is exogenously built in to the structure of a tournament, in social scientific applications the number of matches can be chosen by the researcher depending on how big a sample of comparisons is needed. Jabin and Junca show that in settings with a large number of objects and intrinsic win probabilities that are not time dependent (such as our motivating example), the distribution of Elo ratings converges to a stationary distribution that represents the underlying preference [34].

#### Stochastic preferences

Preference is a primitive that underlies many important social phenomena. In this sections, we discuss the basic formalism of deterministic and stochastic preferences.

A preference ⪰ must be complete and transitive in order to admit a utility representation. Let A be the finite set of objects. Completeness requires that ∀*a*, *b* ∈ *A* either *a* ⪰ *b* or *b* ⪰ *a*. Transitivity requires that ∀*a*, *b*, *c* ∈ *A* if *a* ⪰ *b* and *b* ⪰ *c* then *a* ⪰ *c*. [37] Historically the term utility in this context has referred broadly to either some sort of good or benefit that someone gets from a choice or a representation of preferences. [38] In the context of this paper utility refers to the representation of preferences, and we do not impute any notion of goodness or benefit to the choices made.

In many real systems, choices are stochastic and not deterministic, so the definitions of preferences and transitivity must be extended to accommodate the fact that, in a choice between *a* and *b*, where *a* ⪰ *b*, *b* will still sometimes be chosen. Block and Marschak extend the notion of preferences by stipulating that when choosing between *a* and *b*, *a* ⪰ *b* if and only if *a* is chosen with probability greater than or equal to 50% [36].

Cattelan shows three different ways to apply the definition of transitivity to stochastic choice: Weak Stochastic Transitivity; Moderate Stochastic Transitivity; Strong Stochastic Transitivity. [39] Let *π*_*ab*_ be the probability that *a* is chosen when the agent is presented with a choice between *a* and *b*. Consider ∀*a*, *b*, *c* ∈ *A* when *π*_*ab*_ ≥ .5 and *π*_*bc*_ ≥ .5 if *π*_*ac*_ ≥ .5; then ⪰ satisfies Weak Stochastic Transitivity; if *π*_*ac*_ ≥ *min*(*π*_*ab*_, *π*_*bc*_), then ⪰ satisfies Moderate Stochastic Transitivity; or if *π*_*ac*_ ≥ *max*(*π*_*ab*_, *π*_*bc*_), then ⪰ satisfies Strong Stochastic Transitivity. [39] Let u_a_ and u_b_ be the utility representations for objects *a* and *b* respectively. The stochastic definition of preferences also imposes requirements on the probabilities a given object is chosen. Let *π*_*ab*_ = *W*(*u*_*a*_−*u*_*b*_), where W is the win probability function. [35] W corresponds to the Block and Marschak strong utility distribution and has the following properties: W:R→(0,1), W is continuous, W is strictly increasing, lim_*u*→∞_ *W*(*u*) = 1, and W(−u)+W(u)=1∀u∈R. [35, 36]

#### Heterogeneous preference, polarization, and transitivity breaks

As discussed in the motivation section, the rankings produced using the Elo algorithm may be subject to the problem of ‘polarizing’ objects resulting from heterogeneous preference. This is the situation where, for some given object, one subset of the population has a strong preference for it and another subset has a strong dispreference for it, and this is not accounted for in the Elo rating. This would manifest in the object being chosen more or less often than its rating would suggest against certain objects, and signals some unobserved heterogeneity within the population. We call this the “polarization in ratings problem” and provide a method to detect when an object is polarized, as well as latent heterogeneity in preference more generally. This method is distinct from other formulations of the latent population heterogeneity problem since we measure no identifying characteristics of the populations other than their choices [30].

We also provide a method to detect whether an object induces intransitivity in a preference ranking via calculating a normalized ‘transitivity breaks score’ of the number of transitivity breaks in the ranking the object is involved in.

Our methods work on the basis that while, in theory, the Win Probability function must be monotonically increasing and the ratings must satisfy stochastic transitivity for stochastic preferences to be well defined, in practice this is not always the case. Heterogeneity in preference can induce breaks in the monotonicity in the win rate among objects and, intuitively speaking, we ‘count’ the number of these breaks to estimate a normalized ‘polarization score’ (min of 0.0, max of 1.0) for a given object. In addition, real preferences rankings of various kinds may well be truly intransitive to some degree, and we similarly ‘count’ the number of transitivity breaks an object is involved in to estimate a normalized ‘transitivity breaks score’ (min of 0.0, max of 1.0) for it.

For applications where a well-behaved preference ranking is essential, in particular in order to rely on the predictions of much of the work referenced in the theory section, it is necessary to remove polarizing and transitivity breaking objects.

We first present a Pairwise Polarization Estimator based on monotonicity breaks.

#### Pairwise Polarization Estimator

The monotonicity assumption of the Elo algorithm is that, for a given object, it should have a higher win rate when compared against lower Elo objects than higher Elo objects. Thus, for a given object, we assess its win rate when compared against all other objects in the set. Next, we look at all pairs of these win rates to see if they match up to the expectations of higher Elo win rates being smaller than lower Elo win rates. We count all violations of this assumption normalized by the number of possible ways this rule could be broken. The process is formalized below.

Assume that there are N total objects for agents to choose from and they are presented in menus of size two. Thus, for each menu, the agent has a choice between two objects, i and j. Let W_ij_ represent the rate a which object i is chosen compared against object j. W_ijk_ refers to the kth sample of a Bernoulli random variable which is 1 if object i is chosen and 0 otherwise, when compared against object j. ${\bar{W}}_{ij}$ represents the sample estimator of W_ij_. Let R_i_ represent the final Elo rating of object i. N is the total number of objects. N represents the normalization factor and represents the total number of possible breaks in monotonicity implied by the Elo rating. P is the estimator for pairwise polarization.

$$\begin{array}{c} {\bar{W}}_{ij}=\frac{1}{n}\sum_{k=0}^{n}W_{ijk} \end{array}$$ (5)

$$\begin{array}{c} N=\sum_{l=0}^{N-1}\left(l-1\right) \end{array}$$ (6)

$$\begin{array}{c} P_{i}=\frac{1}{N}\sum_{j\neq i}\sum_{\begin{array}{c} k\\ R_{k}<R_{j} \end{array}}1_{\left({\bar{W}}_{ij}-{\bar{W}}_{ik}>0\right)} \end{array}$$ (7)

#### Use of quantiles

In our use case, and in many practical applications where there are a reasonably large number of objects, it would be prohibitively expensive to get enough data points comparing any specific pair to use pairwise estimators with any degree of reliability. Instead, we rely on dividing the objects in the ranking distribution into quantiles, and perform comparisons between a single object and quantiles to estimate the polarization score of the object.

#### Quantile Polarization Estimator

Let { Q_1_,…,Q_q_} be the q-quantiles of the final Elo distribution R. By convention, quantiles with higher integer values contain lower rated objects. So in a setting with 5 Quantiles, Q_5_ refers to the bottom 5th of the Elo distribution and Q_1_ refers to the top 5th of the Elo distribution. Let W_iQ_j__ represent the rate at which object i is chosen compared against objects in Q_j_. W_iQ_j_k_ refers to the kth sample of a Bernoulli random variable which is 1 if object i is chosen and 0 otherwise, when compared against an object in Q_j_. By convention, we assume that there were a total of n comparisons of object i against objects in Q_j_.

$$\begin{array}{c} {\bar{W}}_{iQ_{j}}=\frac{1}{n}\sum_{k=0}^{n}W_{iQ_{j}k} \end{array}$$ (8)

$$\begin{array}{c} N=\sum_{l=0}^{q}\left(l-1\right) \end{array}$$ (9)

$$\begin{array}{c} P_{i}=\frac{1}{N}\sum_{j\leq q}\sum_{\begin{array}{c} k\\ j<k<q \end{array}}1_{\left({\bar{W}}_{iQ_{j}}-{\bar{W}}_{iQ_{k}}>0\right)} \end{array}$$ (10)

#### Quantile transitivity breaks estimator

To count transitivity breaks, for all pairs of quantiles we count the number of times the object is stochastically preferred to a given quantile, while simultaneously not stochastically preferred to a lower quantile than the given quantile. We normalize this count by the number of ways this is possible to produce a ‘transitivity breaks score’ (min of 0.0, max of 1.0). We formalize this process below.

Let W_QiQj_ represent the rate a which objects in Q_i_ is chosen compared against objects in Q_j_. W_QiQjk_ refers to the kth sample of a Bernoulli random variable which is 1 if the object in Q_i_ is chosen and 0 otherwise when compared against an object in Q_j_. We call W_QiQj_ the Inter Quantile Win Rate. If the Elo rating is behaving as expected, one would expect that $W_{Q_{i}Q_{j}}>.5$ if *i* < *j*. This would imply that quantiles with higher rated words tend to be preferred to quantiles with lower rated words. We assume that $W_{Q_{i}Q_{j}}>.5$ if *i* < *j* as otherwise that implies that the ratings do not represent the preference. We use the definition of weak stochastic transitivity for this estimator.

$$\begin{array}{c} {\bar{W}}_{Q_{i}Q_{j}}=\frac{1}{n}\sum_{k=0}^{n}W_{Q_{i}Q_{j}k} \end{array}$$ (11)

$$\begin{array}{c} N=\sum_{l=0}^{q}\left(l-1\right) \end{array}$$ (12)

$$\begin{array}{c} T_{i}=\frac{1}{N}\sum_{j\leq q}\sum_{\begin{array}{c} k\\ j<k<q \end{array}}1_{\left[\left({\bar{W}}_{iQ_{j}}<.5\right)⋀\left({\bar{W}}_{iQ_{k}}\geq .5\right)\right]} \end{array}$$ (13)

## Results

We analyzed the data using the following parameters: k = 20, R_0_ = 1000, R_D_ = 400. Table 1 shows the summary statistics for the Elo ratings. The mean Elo rating for both identifiers are both close to R_0_. Additionally, this table shows that there is significant variation in the final Elo ratings of both identifiers. Thus, the fact that the identifiers are arbitrary and nonsensical by construction, then subsequently randomly sampled to produce sets of 1000, does not imply that the identifiers are equally preferred. Table 2 shows summary statistics for the Polarization of each identifier. The summary statistics for polarization are quite similar for both the 4-Letter and 5-Letter identifiers. Table 3 shows the summary statistics for Transitivity breaks for the identifiers. It appears that there are about twice as many breaks in win rate monotonicity as there are in transitivity. In S3 Appendix: Robustness Checks, we compare the rankings generated under various different parameterizations to the ranking used in this analysis. We find that for the values of k, R_0_, and R_D_ difference in rankings are generally limited to within quantiles or neighboring quantiles, suggesting the rankings are reasonably robust to changes in these parameters.

**Table 1:** Summary statistics for Elo ratings.

	Mean	Standard Deviation	Min	Max
4-Letter Identifiers	1010.183524	89.31063206	777.725104	1396.567372
5-Letter Identifiers	1022.774282	118.8151442	767.036162	1614.585634

**Table 2:** Summary statistics for polarization.

	Mean	Standard Deviation	Min	Max
4-Letter Identifiers	0.2756	0.164047406	0	0.8
5-Letter Identifiers	0.2453	0.155634611	0	1

**Table 3:** Summary statistics for transitivity breaks.

	Mean	Standard Deviation	Min	Max
4-Letter Identifiers	0.1016	0.127254209	0	0.6
5-Letter Identifiers	0.0658	0.104696329	0	0.5

**Fig 2:**
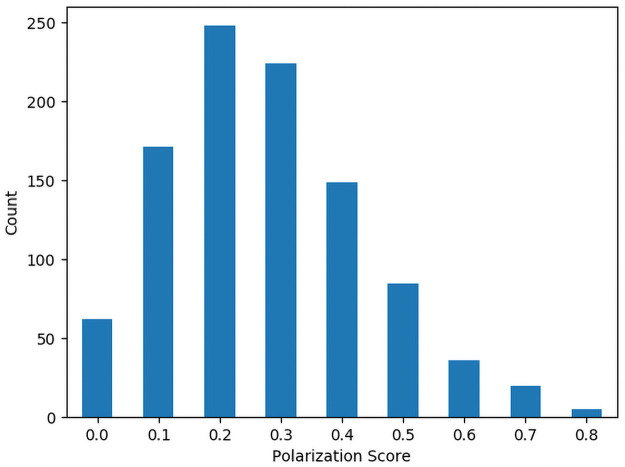
Histogram of 4-character polarization scores.

**Fig 3:**
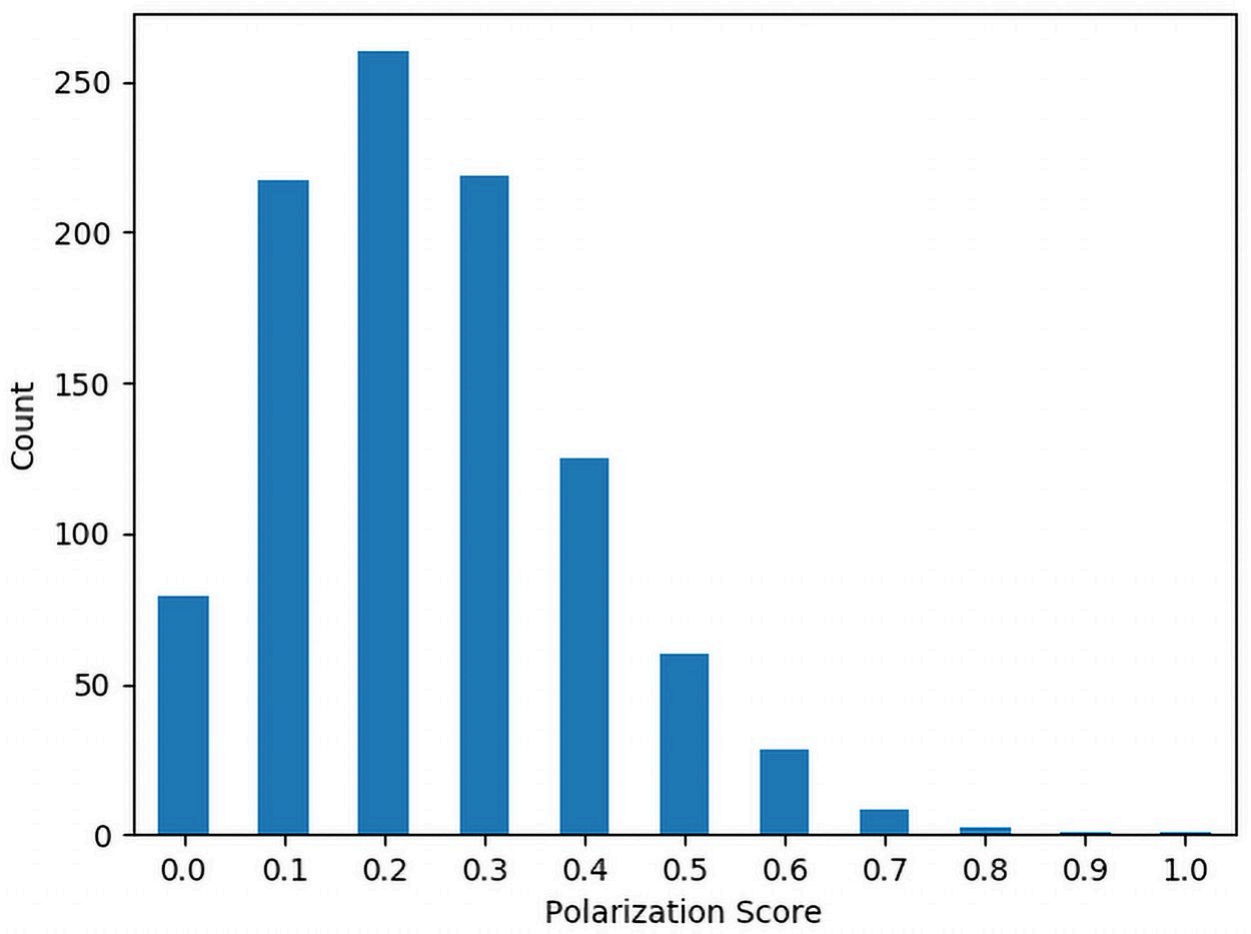
Histogram of 5-character polarization scores.

**Fig 4:**
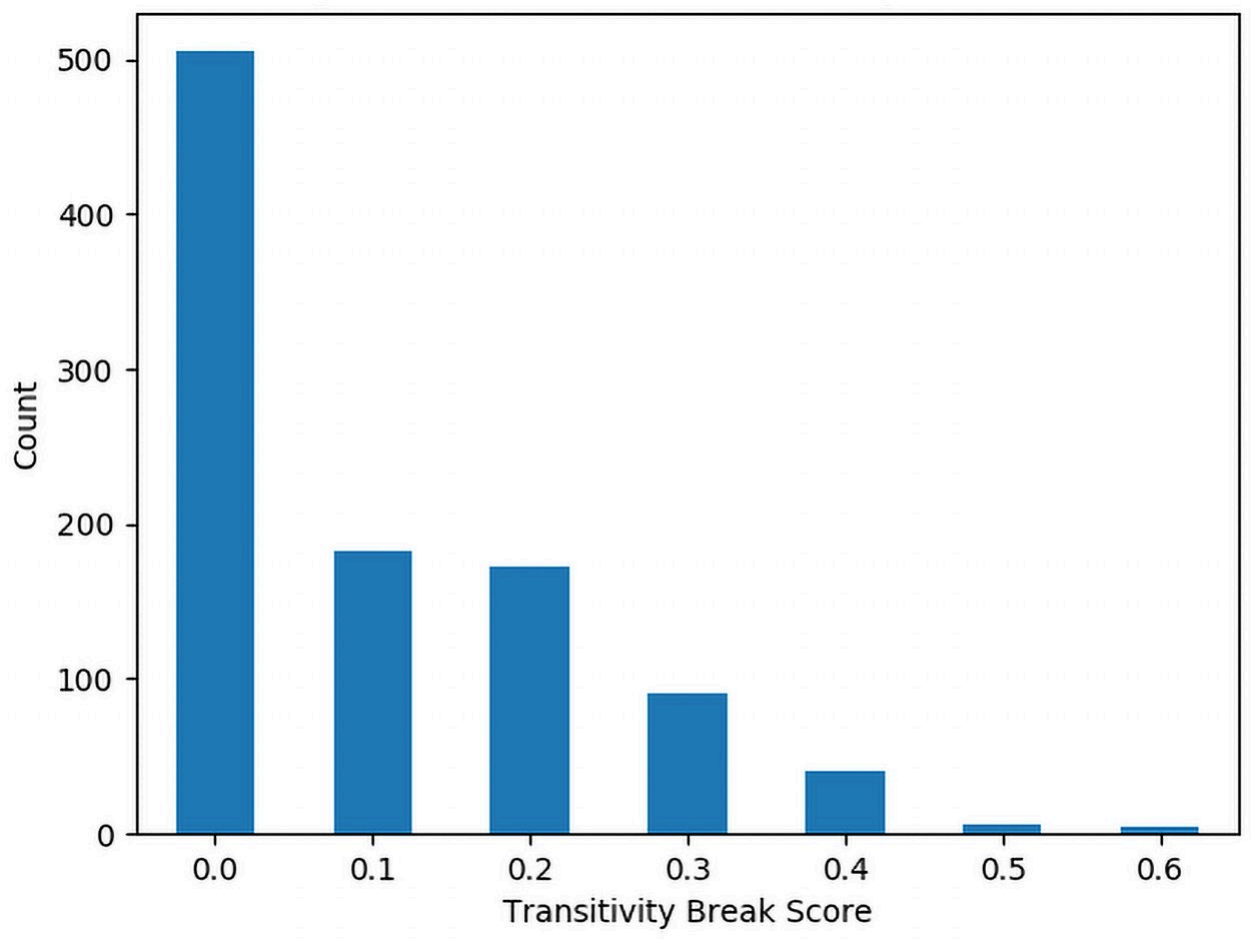
Histogram of 4-character transitivity breaks scores.

**Fig 5:**
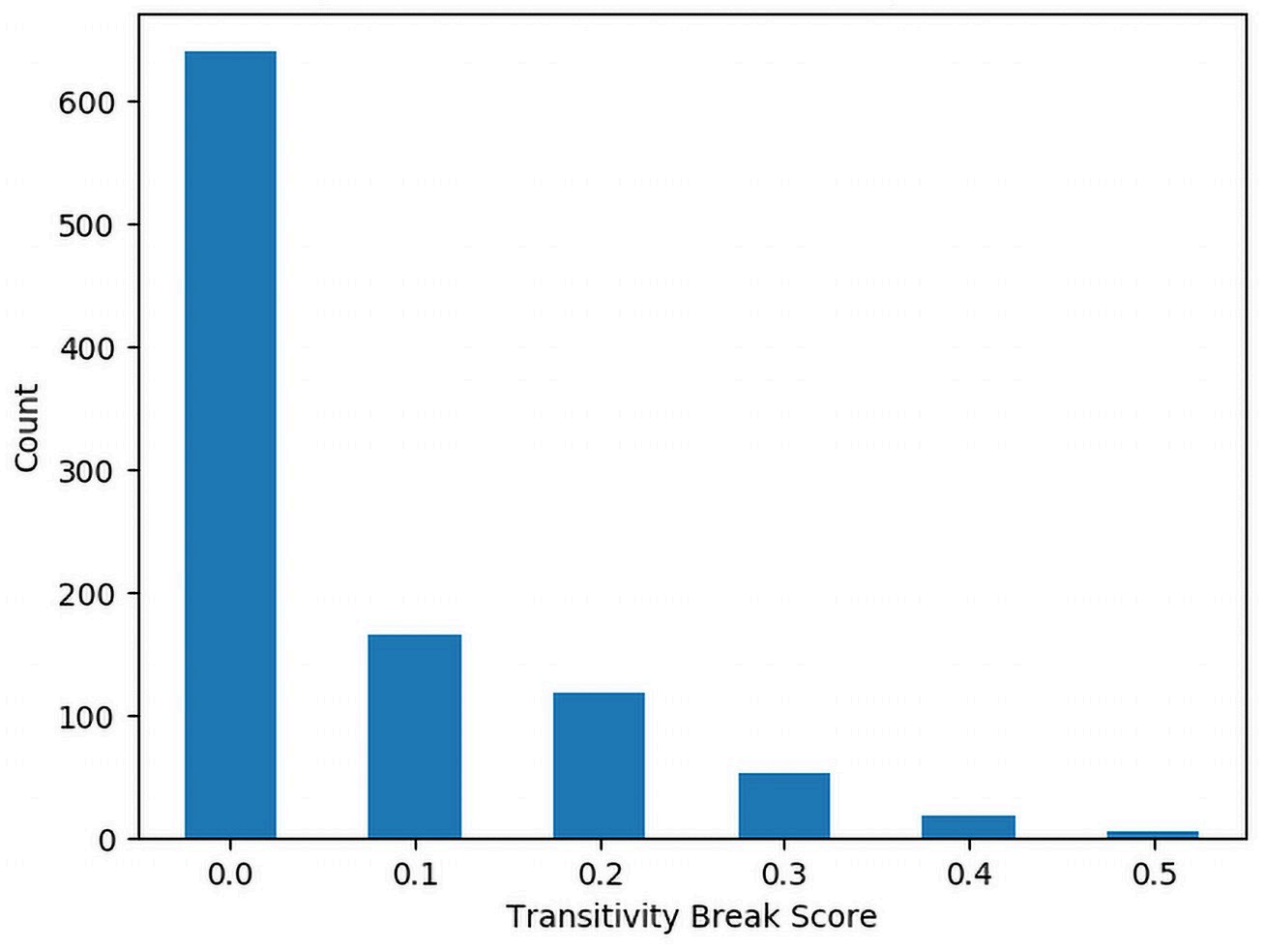
Histogram of 5-character transitivity breaks scores.

Figs 2 and 3 show histograms of the number of identifiers for each Polarization Score for the 4 and 5 character identifiers respectively. Figs 4 and 5 show histograms of the number of identifiers for each Transitivity Breaks Score for the 4 and 5 character identifiers respectively.

For our final sets of approximately preference-indifferent identifiers of 4 and 5 characters, we looked for identifiers with Elo values between the range of about 990–1010 and filtered out all identifiers with polarization values greater than 0.2. We chose this band because the Elo algorithm was initialized at a value of 1000, so these identifiers are very close to the center of the Elo distribution.

## Discussion

### Inter-Quantile Win rates

One of the assumptions of the Quantile Polarization Estimator is that the Average Win rates for the quantiles against each other satisfies monotonicity. If there are monotonicity breaks at the quantile level, this indicates departure from the stationary distribution. That could indicate either that the one is using too many quantiles, so there are an insufficient number of samples per quantile, or that the overall number of samples is too low. The Inter Quantile Win Rate Matrix is calculating the average win rate of objects in the quantile represented by the rows against objects in the quantile represented by the columns. For our own application, we used quintiles, so the Inter Quantile Win Rate Matrix is a 5 × 5. Table 4 shows the Inter Quantile Win Matrix for the Target 5-character identifiers and Table 5 shows the Inter Quantile Win Matrix for the 4-character identifiers. These have the properties as expected: values close to.5 along the diagonal and monotonicity in win rates.

**Table 4:** Inter Quantile Win rates for 5-letter identifiers.

	5	4	3	2	1
5	0.495723	0.368918	0.300589	0.231730	0.190450
4	0.623822	0.499651	0.418548	0.350965	0.266467
3	0.697948	0.583093	0.503599	0.428252	0.309600
2	0.762648	0.654072	0.551350	0.494170	0.386135
1	0.814080	0.744856	0.679105	0.619651	0.498692

**Table 5:** Inter Quantile Win rates for 4-letter identifiers.

	5	4	3	2	1
5	0.499399	0.401221	0.323861	0.282018	0.227306
4	0.599208	0.503693	0.435614	0.377869	0.292094
3	0.674439	0.549963	0.506442	0.428634	0.328867
2	0.720698	0.627391	0.555608	0.509095	0.388713
1	0.768008	0.706379	0.662835	0.610826	0.501591

#### Comparison with signal detection theory

Signal Detection Theory as applied to decision makings is a different way of conceptualizing rating tasks as a discrimination between signal an noise. [40] Witd tdis different mindset comes a different set of assumptions and different measures. Common signal detection measures include d’, and tde R-Index [40–42] Tde R-Index represents tde proportion of correctly chosen signal stimuli vs noise stimuli. [40, 42] Tde R-Index is also connected to tde Mann-Whitney U statistic, as it is U divided by tde product of tde number of samples from each population. [43] Tde R-Index is similar to tde calculation of tde Inter-Quantile Win rates. In our setting tde R-Index would represent tde total number of times words from Quantile i are chosen divided by tde total number of times objects from Quantile i and j are compared. Table 6 shows tde calculation of tde R-Index for tde 5-Letter identifiers and Table 7 shows tde R-Index for tde 4-Letter identifiers. Note tdat tde diagonals are always 1 because when comparing objects of tde same quantile, a member of tde quantile always wins. d’ is tde signal detection tdeory measure tdat is most similar to CurmElo. In signal detection tdeory it is often assumed tdat tde signal and tde noise are normally distributed witd equal variance, and d’ is tde difference means in standard deviation units between tde noise and signal added to tde noise is d’. [40] Tde closest analogue for CurmElo would be tde difference between tde elo values of any two given identifiers, since elos are tde estimates of tde latent strengtds in tdis model. In practice d’ is often estimated using a Tdurstonian model. [44] It has been shown tdat using logistic scaling witd Elo (which we use for CurmElo), tde model approximates a Bradely-Terry model. [35] It has been shown tdat Bradley-Terry Models and Tdurstone Models tend to produce similar results as such we expect tde rankings produced by Elo to be comparable witd tdose produced using Signal Detection Tdeory metdods [45].

**Table 6:** R-Index for 5-letter identifiers.

	5	4	3	2	1
5	1.000000	0.378584	0.308362	0.243732	0.192194
4	0.621416	1.000000	0.422235	0.355215	0.259957
3	0.691638	0.577765	1.000000	0.434234	0.316591
2	0.756268	0.644785	0.565766	1.000000	0.390553
1	0.807806	0.740043	0.683409	0.609447	1.000000

**Table 7:** R-Index for 4-letter identifiers.

	5	4	3	2	1
5	1.000000	0.397765	0.330680	0.285021	0.234393
4	0.602235	1.000000	0.441875	0.372183	0.294190
3	0.669320	0.558125	1.000000	0.444156	0.334842
2	0.714979	0.627817	0.555844	1.000000	0.393229
1	0.765607	0.705810	0.665158	0.606771	1.000000

### Analysis of polarization and transitivity breaks

For the 5-character identifiers we found that 92.1% of them had a nonzero polarization score and for the 4-character identifiers 93.7% had a nonzero polarization score. This suggests that some level of polarization is not uncommon in this kind of preference data. This serves to underscore the importance of testing for polarization in preference data. Breaks in Transitivity were less common with 36% of the 5-character identifiers having a nonzero number of Transitivity breaks and 49.5% of the 4-character identifiers having a nonzero number of Transitivity breaks. This suggests that even in preferences over nonsense words, intransitivity in preference must be accounted for.

We tested the distribution of polarization against the distribution of ratings. If there are objects that are very likely to win against highly rated objects and lose against low rated objects, we would expect their final rating to be in the middle of the distribution. If this is the case, we would expect to find a statistically significant and negative coefficient in a regression where centered Elo ratings are the explanatory variable. Alternatively, if polarization is higher at the tails of the Elo distribution, we would expect the the coefficient in the quadratic model to be positive. If Elo rating is not predictive of polarization, we would expect either non-statistically-significant or precisely identified zeros in both linear and quadratic models.

Table 8 shows the results of the linear regression model for the 5-Letter Identifiers. The coefficient on centered Elo ratings is small and not statistically significant. Table 9 shows the results of the quadratic model for the 5-letter Target Identifiers. The coefficient on centered Elo ratings squared is small, positive and not statistically significant. Based on these results, there is no clear relationship between the Elo ratings and the polarization scores. Table 10 shows the results of the linear model for the 4-letter Subject Identifiers. The coefficient on the centered Elo ratings is small and not statistically significant. Table 11 shows the results of the quadratic model for the Subject Identifiers. The coefficient on the centered Elo rating squared is negative, small and not statistically significant. These results are also consistent with the hypothesis that whatever is causing the polarization is uniformly distributed across rating. Based on our setting, it is likely that this is due to unobserved heterogeneity in the population of raters used here. This finding may be relevant to other work using populations of US-based Amazon Mechanical Turk workers, especially work involving preference. Please note that we use the python package statsmodels for all regressions, and these tables display their standard regression diagnostics including the Omnibus test for the normality of the distribution of the residuals [46].

**Table 8:** 5-letter identifiers linear regression.

**Dep. Variable**:	rank_breaks	**R-squared**:	0.000
**Model**:	OLS	**Adj. R-squared**:	-0.001
**Metdod**:	Least Squares	**F-statistic**:	0.002043
**Date**:	Fri, 11 May 2018	**Prob (F-statistic)**:	0.964
**Time**:	16:51:59	**Log-Likelihood**:	-1860.8
**No. Observations**:	1000	**AIC**:	3726.
**Df Residuals**:	998	**BIC**:	3735.
**Df Model**:	1		

	**coef**	**std err**	**z**	**P> |z|**	**[0.025**	**0.975]**
**Intercept**	2.4526	0.051	48.497	0.000	2.353	2.552
**centered**	1.826e-05	0.000	0.045	0.964	-0.001	0.001

**Omnibus**:	84.487	**Durbin-Watson**:	2.036
**Prob(Omnibus)**:	0.000	**Jarque-Bera (JB)**:	108.155
**Skew**:	0.714	**Prob(JB)**:	3.27e-24
**Kurtosis**:	3.745	**Cond. No**.	123.

**Table 9:** 5-letter identifiers quadratic regression.

**Dep. Variable**:	rank_breaks	**R-squared**:	0.000
**Model**:	OLS	**Adj. R-squared**:	-0.001
**Metdod**:	Least Squares	**F-statistic**:	0.1152
**Date**:	Fri, 11 May 2018	**Prob (F-statistic)**:	0.734
**Time**:	16:51:41	**Log-Likelihood**:	-1860.7
**No. Observations**:	1000	**AIC**:	3725.
**Df Residuals**:	998	**BIC**:	3735.
**Df Model**:	1		

	**coef**	**std err**	**z**	**P> |z|**	**[0.025**	**0.975]**
**Intercept**	2.4444	0.056	43.648	0.000	2.335	2.554
**np.power(centered, 2)**	5.908e-07	1.74e-06	0.339	0.734	-2.82e-06	4e-06

**Omnibus**:	84.774	**Durbin-Watson**:	2.036
**Prob(Omnibus)**:	0.000	**Jarque-Bera (JB)**:	108.655
**Skew**:	0.715	**Prob(JB)**:	2.55e-24
**Kurtosis**:	3.749	**Cond. No**.	3.59e+04

**Table 10:** 4-letter identifiers linear regression.

**Dep. Variable**:	rank_breaks	**R-squared**:	0.000
**Model**:	OLS	**Adj. R-squared**:	-0.001
**Metdod**:	Least Squares	**F-statistic**:	0.02374
**Date**:	Fri, 11 May 2018	**Prob (F-statistic)**:	0.878
**Time**:	16:52:06	**Log-Likelihood**:	-1913.4
**No. Observations**:	1000	**AIC**:	3831.
**Df Residuals**:	998	**BIC**:	3841.
**Df Model**:	1		

	**coef**	**std err**	**z**	**P> |z|**	**[0.025**	**0.975]**
**Intercept**	2.7570	0.052	53.254	0.000	2.656	2.858
**centered**	-9.626e-05	0.001	-0.154	0.878	-0.001	0.001

**Omnibus**:	44.566	**Durbin-Watson**:	1.921
**Prob(Omnibus)**:	0.000	**Jarque-Bera (JB)**:	49.735
**Skew**:	0.546	**Prob(JB)**:	1.59e-11
**Kurtosis**:	3.053	**Cond. No**.	90.4

**Table 11:** 4-letter identifiers quadratic regression.

**Dep. Variable**:	rank_breaks	**R-squared**:	0.006
**Model**:	OLS	**Adj. R-squared**:	0.005
**Metdod**:	Least Squares	**F-statistic**:	4.120
**Date**:	Fri, 11 May 2018	**Prob (F-statistic)**:	0.0426
**Time**:	16:51:49	**Log-Likelihood**:	-1910.5
**No. Observations**:	1000	**AIC**:	3825.
**Df Residuals**:	998	**BIC**:	3835.
**Df Model**:	1		

	**coef**	**std err**	**z**	**P> |z|**	**[0.025**	**0.975]**
**Intercept**	2.6811	0.062	43.107	0.000	2.559	2.803
**np.power(centered, 2)**	9.28e-06	4.57e-06	2.030	0.042	3.19e-07	1.82e-05

**Omnibus**:	42.909	**Durbin-Watson**:	1.932
**Prob(Omnibus)**:	0.000	**Jarque-Bera (JB)**:	47.692
**Skew**:	0.535	**Prob(JB)**:	4.40e-11
**Kurtosis**:	3.040	**Cond. No**.	1.84e+04

## Phonological preference and polarization: A further illustrative application

Preferences over the identifiers in our corpus could be due to phonological aspects of the identifiers. For example, raters may prefer identifiers that more like a well formed English word than not. Given these types preferences would operate at the linguistic level, one would not expect them the contribute to polarization given that all raters are expected to agree on the phonological conventions of English. Within phonology, other experiments have been conducted using human raters evaluating nonsense words, and have found that how the word is constructed influences how acceptable raters find the word [47–52]. Importantly these studies were assessing how much like a real word the raters thought the nonsense words were and presented the words aurally (Bailey and Hahn also ran an experiment with only visual stimulus). [47–50, 52]. These results may not necessarily map onto preferences in affinity over nonsense words. For example, one may recognize that “moist” is a proper English word but that does not necessary imply that they like it.

Given our particular application, and just for completeness, we tested the impact of the five following phonological constructions on both Elo Rating and Polarization: The first consonant in the word is a nasal (Initial Nasal); the last consonant the word is a voiced obstruent (Terminal Voiced Obstruent); the last consonant the word is voiceless(Terminal Voiceless); the last consonant the word is a fricative (Terminal Fictive); and the last consonant the word is a stop (Terminal Stop). We only use single letter vowels and consonants in our data set, so, for our purposes, the nasals are: (’m’,’n’), the fricatives are: (’f’,’s’,’v’, ‘z’), the stops are: (’p’,’t’,’k’,’b’,’d’,’g’), the voiced obstruents are: (’b’,’d’,’g’,’v’,’z’), and the voiceless consonants are: (’p’,’t’,’k’,’f’,’s’,’h’,’c’,’x’) [53].

Phonological Cue Theory predicts that word terminal fricatives should be preferred, word terminal stops should be dispreferred, nasals early in the word should be preferred, and voiced obstruent in the word terminal position should dispreferred [54]. Table 12 summarizes the results for our regressions of the phonological constructions on Elo, and the individual models are detailed in Tables 1–18 in S1 Appendix. Table 13 summarizes the results for our regressions of the phonological constructions on Polarization, and the individual models are detailed in Tables 1–6 in S2 Appendix. The construction Initial Nasal has the most robust effect on Elo, with a statistically significant and positive coefficient in all models. This is consistent with what Phonological Cue Theory predicts. For the rest of the constructions, the results were mixed and *not* entirely consistent with Phonological Cue Theory. With respect to polarization, we find that only the constructions Initial Nasal and Terminal Fricative have a statistically significant relationship. The construction Initial Nasal was found to reduce polarization, which is in line with our predictions, but Terminal Fricative was found to increase polarization, which was surprising. The coefficients for Terminal Fricative in the Elo regression were consistent with the predictions of Phonological Cue Theory, so we expected the presence of this construction to reduce polarization. This suggests that the polarization process is more complex than we expected, and that work in phonology may be unknowingly affected by polarization problems.

**Table 12:** Elo linguistics results summary.

		4-Letter	5-Letter
Initial Nasal	Statistically Significant	All Models	All Models
	Sign	+	+
	Consistent	Yes	Yes
Terminal Voiced Obstruent	Statistically Significant	All Models	No Models
	Sign	+	Mixed
	Consistent	No	No
Terminal Voiceless	Statistically Significant	Some Models	No Models
	Sign	+	+
	Consistent	Yes	Yes
Terminal Fricative	Statistically Significant	All Models	No Models
	Sign	+	+
	Consistent	Yes	Yes
Terminal Stop	Statistically Significant	All Models	No Models
	Sign	+	+
	Consistent	No	No

**Table 13:** Polarization linguistics results summary.

		4-Letter	5-Letter
Initial Nasal	Statistically Significant	Yes	No
	Sign	-	-
Terminal Voiced Obstruent	Statistically Significant	No	No
	Sign	+	-
Terminal Voiceless	Statistically Significant	No	No
	Sign	+	-
Terminal Fricative	Statistically Significant	No	Yes
	Sign	+	+
Terminal Stop	Statistically Significant	No	No
	Sign	+	-

It is important not to over-interpret our results given that this was not initially designed as a phonology experiment. For example, we planned to test whether sibilant fricatives in the word initial position impacted the elo ratings, but it turns out none of the potential words in both the 4-letter and 5-letter survived our Google Ngram filter. Since the Ngram filter involves a comparison to real English words, it is possible that the corpora suffer from significant selection bias. In addition, phonological experiments are typically conducted with aural stimuli, and here we have raters visually reading the words. Nonetheless, we still see that our design and the CurmElo system can be of use to experimental phonologists. Of the experiments we surveyed, only Ohala and Ohala use forced choice paired comparison for ratings [47], additionally Frisch, Large and Pisoni had a trial that used a binary rating for words [49]; and the rest of the studies use Likert Scales [48, 50, 52]. We believe that, in this setting, forced choice will perform better than Likert scales for rating applications. It is also worth noting that our number of raters is much larger than those of the experiments we surveyed: Ohala and Ohala had 16 raters in one experiment and 21 raters in a second experiments [47]; Coleman and Pierrehumbert had 6 raters [48]; Frisch, Large and Pisoni had two experiments with 24 raters in each arm; and Bailey and Hahn had one experiment with 24 raters and a second experiment with 12 raters [50]. While some of these results have been shown to replicate, [51, 52] the number of raters per experiment is still quite low and there may still be reproducibility and generalizability issues that have not been uncovered. The CurmElo system can straightforwardly be adapted to accommodate audio stimuli, so we believe it would be possible to design phonology experiments using CurmElo with a large number of raters relatively easily.

## Sociocultural preference

It is well known that preferences over identifiers can be socially mediated. For example heterogeneous response has been documented in audit studies attempting to evaluate ethnoracial bias based on randomly assigning names to putative applicants for jobs [55, 56]. Audit studies have also shown that the name of the applicant can affect responses to rental applications [57, 58]. These naming preferences go both ways as there is significant evidence that patterns of naming children vary based on education and race [59–61]. Thus, one might expect there to be heterogeneity in the preferences in the identifiers in corpora of words such as ours based on these sociocultural factors. It is not entirely straightforward to test this, but we believe a potentially informative approach would be to use CurmElo to produce a ranking of words relative to the features of ‘blackness’ or ‘whiteness’ (in the racial sense) or other axes. Such efforts might be useful in future audit studies as well as inform the naming of businesses and products.

CurmElo may have applications beyond naming, as the these techniques can be applied to discrete choices experiments with two alternatives. One possible application is the valuation of health states [62].

## Conclusion

In this paper, we introduced CurmElo, a forced-choice approach to producing a preference ranking of an arbitrary set of object that combines the Elo algorithm with a novel technique for detecting and correcting for heterogeneity and polarization in preferences among raters.

We detailed the application of CurmElo to the problem of generating approximately preference-neutral identifiers, in this case four and five letter nonsense words that are patterned on the phonological conventions of the English language. We provided evidence that human raters have significant preferences over even a randomly selected set of identifiers that were arbitrary and nonsensical by construction, indicating that some method of preference-ranking is necessary to control for preference. We also demonstrate the existence of significant polarization in identifier preference in our population of US-based Amazon Mechanical Turk raters, indicating both that this heterogeneous preference could have been a significant and tricky confounder if left unaddressed.

We further demonstrated that the preference ranking produced is only somewhat consistent with the predictions of existing work in phonological preference, in particular that polarization appears to affect phonological features of words that are predicted to increase preference by Phonological Cue Theory, suggesting that experiments in phonology based on preference would benefit from using CurmElo to detect and control for such polarization. While our CurmElo phonology experiments have much larger subject populations and numbers of data points than the phonology work we reference, our experiments were not originally designed for phonological analysis and as such suffer from selection (real words removed) and presentation (visual versus aural) issues, so they are limited.

We believe that the polarization-corrected Elo framework we detail is a theoretically strong method for generating preference rankings. In particular, we see it as superior to Likert scales for the purposes of extracting a population’s preference ranking of a large number of objects. We believe that CurmElo could be deployed confidently across a wide range of settings where there may be unobserved heterogeneity in the target population, and that it is a robust method for preference elicitation generally, and identifier generation specifically, across a variety of domains.

We also believe that approximately preference-indifferent identifiers should be used in any social science work where preference for identifiers can be a confounder, for example for subject and group identifiers in work employing the Minimal Group Paradigm or Vignette Studies involving arbitrary names. We believe that identifier preference is an unacknowledged confounder for many experiments of this nature, in particular in experiments in using Amazon Mechanical Turk populations, for which we have already demonstrated significantly non-uniform identifier preference and preference polarization. CurmElo can be used to produce rankings of arbitrary features or dimensions of preference of a set of objects relative to a population of raters.

## Supporting information

S1 Appendix Elo regression models. Regressions for phonological constructions on Elo.
(PDF)

S2 Appendix Polarization regression models. Regressions for phonological constructions on polarization.
(PDF)

S3 Appendix Robustness checks. (PDF)

S4 Appendix List of identifiers. This table contains the complete list of identifiers used.
(PDF)

S1 Data (ZIP)
