# Supplementary material for: CurmElo: The theory and practice of a forced-choice approach to producing preference rankings
Source: PLoS One. 2021 May 27;16(5):e0252145. doi: 10.1371/journal.pone.0252145 (PMC8158949; doi:10.1371/journal.pone.0252145)
Supplement: S1 Appendix — Regressions for phonological constructions on Elo. (PDF) [file pone.0252145.s001.pdf]

**S1 Appendix: Elo Regression Models. Regressions for Phonological Constructions on Elo.**

**Table 1. 5-Letter Identifiers Elo vs Initial Nasal**

| | | | |
|---|---|---|---|
| **Dep. Variable:** | elo | **R-squared:** | 0.015 |
| **Model:** | OLS | **Adj. R-squared:** | 0.014 |
| **Method:** | Least Squares | **F-statistic:** | 15.07 |
| **Date:** | Wed, 29 Aug 2018 | **Prob (F-statistic):** | 0.000110 |
| **Time:** | 19:47:17 | **Log-Likelihood:** | -6188.3 |
| **No. Observations:** | 1000 | **AIC:** | 1.238e+04 |
| **Df Residuals:** | 998 | **BIC:** | 1.239e+04 |
| **Df Model:** | 1 | | |

| | **coef** | **std err** | **z** | **P> \|z\|** | **[0.025** | **0.975]** |
|---|---|---|---|---|---|---|
| **const** | 1016.3492 | 4.059 | 250.385 | 0.000 | 1008.393 | 1024.305 |
| **initial_nasal** | 40.1567 | 10.343 | 3.883 | 0.000 | 19.885 | 60.428 |

| | | | |
|---|---|---|---|
| **Omnibus:** | 127.444 | **Durbin-Watson:** | 0.033 |
| **Prob(Omnibus):** | 0.000 | **Jarque-Bera (JB):** | 200.368 |
| **Skew:** | 0.864 | **Prob(JB):** | 3.09e-44 |
| **Kurtosis:** | 4.351 | **Cond. No.** | 2.81 |

**Table 2. 5-Letter Identifiers Elo vs Terminal Voiceless Consonant**

| | | | |
|---|---|---|---|
| **Dep. Variable:** | elo | **R-squared:** | 0.001 |
| **Model:** | OLS | **Adj. R-squared:** | 0.000 |
| **Method:** | Least Squares | **F-statistic:** | 1.116 |
| **Date:** | Wed, 29 Aug 2018 | **Prob (F-statistic):** | 0.291 |
| **Time:** | 19:47:17 | **Log-Likelihood:** | -6195.5 |
| **No. Observations:** | 1000 | **AIC:** | 1.239e+04 |
| **Df Residuals:** | 998 | **BIC:** | 1.240e+04 |
| **Df Model:** | 1 | | |

| | **coef** | **std err** | **z** | **P> \|z\|** | **[0.025** | **0.975]** |
|---|---|---|---|---|---|---|
| **const** | 1019.6074 | 5.003 | 203.814 | 0.000 | 1009.802 | 1029.412 |
| **terminal_voiceless** | 7.9770 | 7.552 | 1.056 | 0.291 | -6.824 | 22.778 |

| | | | |
|---|---|---|---|
| **Omnibus:** | 124.464 | **Durbin-Watson:** | 0.003 |
| **Prob(Omnibus):** | 0.000 | **Jarque-Bera (JB):** | 189.968 |
| **Skew:** | 0.862 | **Prob(JB):** | 5.61e-42 |
| **Kurtosis:** | 4.261 | **Cond. No.** | 2.45 |

Table 3. **5-Letter Identifiers Elo vs Terminal Voiced Obstruent**

| | | | |
|---|---|---|---|
| **Dep. Variable:** | elo | **R-squared:** | 0.000 |
| **Model:** | OLS | **Adj. R-squared:** | -0.001 |
| **Method:** | Least Squares | **F-statistic:** | 0.009826 |
| **Date:** | Wed, 29 Aug 2018 | **Prob (F-statistic):** | 0.921 |
| **Time:** | 19:47:17 | **Log-Likelihood:** | -6196.0 |
| **No. Observations:** | 1000 | **AIC:** | 1.240e+04 |
| **Df Residuals:** | 998 | **BIC:** | 1.241e+04 |
| **Df Model:** | 1 | | |

| | **coef** | **std err** | **z** | **P> \|z\|** | **[0.025** | **0.975]** |
|---|---|---|---|---|---|---|
| **const** | 1022.9633 | 4.342 | 235.596 | 0.000 | 1014.453 | 1031.474 |
| **terminal_obstruents** | -0.8554 | 8.629 | -0.099 | 0.921 | -17.768 | 16.057 |

| | | | |
|---|---|---|---|
| **Omnibus:** | 121.775 | **Durbin-Watson:** | 0.001 |
| **Prob(Omnibus):** | 0.000 | **Jarque-Bera (JB):** | 183.882 |
| **Skew:** | 0.851 | **Prob(JB):** | 1.18e-40 |
| **Kurtosis:** | 4.230 | **Cond. No.** | 2.55 |

Table 4. **5-Letter Identifiers Elo vs Initial Nasal and Terminal Voiceless Consonant**

| | | | |
|---|---|---|---|
| **Dep. Variable:** | elo | **R-squared:** | 0.016 |
| **Model:** | OLS | **Adj. R-squared:** | 0.014 |
| **Method:** | Least Squares | **F-statistic:** | 8.151 |
| **Date:** | Wed, 29 Aug 2018 | **Prob (F-statistic):** | 0.000308 |
| **Time:** | 19:47:17 | **Log-Likelihood:** | -6187.9 |
| **No. Observations:** | 1000 | **AIC:** | 1.238e+04 |
| **Df Residuals:** | 997 | **BIC:** | 1.240e+04 |
| **Df Model:** | 2 | | |

| | **coef** | **std err** | **z** | **P> \|z\|** | **[0.025** | **0.975]** |
|---|---|---|---|---|---|---|
| **const** | 1013.6635 | 5.129 | 197.632 | 0.000 | 1003.611 | 1023.716 |
| **initial_nasal** | 39.8241 | 10.388 | 3.834 | 0.000 | 19.464 | 60.184 |
| **terminal_voiceless** | 6.8990 | 7.528 | 0.916 | 0.359 | -7.856 | 21.654 |

| | | | |
|---|---|---|---|
| **Omnibus:** | 129.467 | **Durbin-Watson:** | 0.036 |
| **Prob(Omnibus):** | 0.000 | **Jarque-Bera (JB):** | 205.081 |
| **Skew:** | 0.872 | **Prob(JB):** | 2.93e-45 |
| **Kurtosis:** | 4.372 | **Cond. No.** | 3.07 |

**Table 5. 5-Letter Identifiers Elo vs Initial Nasal,Terminal Voiceless Consonant, and Terminal Voiced Obstruent**

| | | | |
|---|---|---|---|
| **Dep. Variable:** | elo | **R-squared:** | 0.016 |
| **Model:** | OLS | **Adj. R-squared:** | 0.013 |
| **Method:** | Least Squares | **F-statistic:** | 5.454 |
| **Date:** | Wed, 29 Aug 2018 | **Prob (F-statistic):** | 0.00101 |
| **Time:** | 19:47:17 | **Log-Likelihood:** | -6187.8 |
| **No. Observations:** | 1000 | **AIC:** | 1.238e+04 |
| **Df Residuals:** | 996 | **BIC:** | 1.240e+04 |
| **Df Model:** | 3 | | |

| | **coef** | **std err** | **z** | **P> \|z\|** | **[0.025** | **0.975]** |
|---|---|---|---|---|---|---|
| **const** | 1012.2185 | 6.751 | 149.930 | 0.000 | 998.986 | 1025.451 |
| **initial_nasal** | 39.8237 | 10.384 | 3.835 | 0.000 | 19.472 | 60.175 |
| **terminal_voiceless** | 8.3442 | 8.710 | 0.958 | 0.338 | -8.726 | 25.415 |
| **terminal_obstruents** | 3.9430 | 9.837 | 0.401 | 0.689 | -15.337 | 23.223 |

| | | | |
|---|---|---|---|
| **Omnibus:** | 130.985 | **Durbin-Watson:** | 0.036 |
| **Prob(Omnibus):** | 0.000 | **Jarque-Bera (JB):** | 208.912 |
| **Skew:** | 0.877 | **Prob(JB):** | 4.32e-46 |
| **Kurtosis:** | 4.392 | **Cond. No.** | 3.67 |

**Table 6. 5-Letter Identifiers Elo vs Terminal Fricative**

| | | | |
|---|---|---|---|
| **Dep. Variable:** | elo | **R-squared:** | 0.001 |
| **Model:** | OLS | **Adj. R-squared:** | -0.000 |
| **Method:** | Least Squares | **F-statistic:** | 1.033 |
| **Date:** | Wed, 29 Aug 2018 | **Prob (F-statistic):** | 0.310 |
| **Time:** | 19:47:17 | **Log-Likelihood:** | -6195.5 |
| **No. Observations:** | 1000 | **AIC:** | 1.240e+04 |
| **Df Residuals:** | 998 | **BIC:** | 1.240e+04 |
| **Df Model:** | 1 | | |

| | **coef** | **std err** | **z** | **P> \|z\|** | **[0.025** | **0.975]** |
|---|---|---|---|---|---|---|
| **const** | 1021.0435 | 4.198 | 243.217 | 0.000 | 1012.815 | 1029.272 |
| **terminal_fricative** | 9.5621 | 9.408 | 1.016 | 0.309 | -8.878 | 28.002 |

| | | | |
|---|---|---|---|
| **Omnibus:** | 123.381 | **Durbin-Watson:** | 0.003 |
| **Prob(Omnibus):** | 0.000 | **Jarque-Bera (JB):** | 187.486 |
| **Skew:** | 0.858 | **Prob(JB):** | 1.94e-41 |
| **Kurtosis:** | 4.248 | **Cond. No.** | 2.70 |

**Table 7. 5-Letter Identifiers Elo vs Terminal Stop**

| | | | |
|---|---|---|---|
| **Dep. Variable:** | elo | **R-squared:** | 0.001 |
| **Model:** | OLS | **Adj. R-squared:** | -0.000 |
| **Method:** | Least Squares | **F-statistic:** | 0.5403 |
| **Date:** | Wed, 29 Aug 2018 | **Prob (F-statistic):** | 0.462 |
| **Time:** | 19:47:17 | **Log-Likelihood:** | -6195.8 |
| **No. Observations:** | 1000 | **AIC:** | 1.240e+04 |
| **Df Residuals:** | 998 | **BIC:** | 1.241e+04 |
| **Df Model:** | 1 | | |

| | **coef** | **std err** | **z** | **P> \|z\|** | **[0.025** | **0.975]** |
|---|---|---|---|---|---|---|
| **const** | 1021.1161 | 4.498 | 227.036 | 0.000 | 1012.301 | 1029.931 |
| **terminal_stop** | 6.0078 | 8.173 | 0.735 | 0.462 | -10.011 | 22.026 |

| | | | |
|---|---|---|---|
| **Omnibus:** | 123.301 | **Durbin-Watson:** | 0.002 |
| **Prob(Omnibus):** | 0.000 | **Jarque-Bera (JB):** | 187.515 |
| **Skew:** | 0.857 | **Prob(JB):** | 1.91e-41 |
| **Kurtosis:** | 4.251 | **Cond. No.** | 2.45 |

**Table 8.** 5-Letter Identifiers Elo vs Terminal Fricative and Terminal Stop

| | | | |
|---|---|---|---|
| **Dep. Variable:** | elo | **R-squared:** | 0.002 |
| **Model:** | OLS | **Adj. R-squared:** | 0.000 |
| **Method:** | Least Squares | **F-statistic:** | 1.042 |
| **Date:** | Wed, 29 Aug 2018 | **Prob (F-statistic):** | 0.353 |
| **Time:** | 19:47:17 | **Log-Likelihood:** | -6195.0 |
| **No. Observations:** | 1000 | **AIC:** | 1.240e+04 |
| **Df Residuals:** | 997 | **BIC:** | 1.241e+04 |
| **Df Model:** | 2 | | |

| | **coef** | **std err** | **z** | **P> \|z\|** | **[0.025** | **0.975]** |
|---|---|---|---|---|---|---|
| **const** | 1017.9530 | 5.301 | 192.025 | 0.000 | 1007.563 | 1028.343 |
| **terminal_fricative** | 12.6527 | 9.949 | 1.272 | 0.203 | -6.848 | 32.153 |
| **terminal_stop** | 9.1710 | 8.641 | 1.061 | 0.289 | -7.766 | 26.107 |

| | | | |
|---|---|---|---|
| **Omnibus:** | 125.877 | **Durbin-Watson:** | 0.005 |
| **Prob(Omnibus):** | 0.000 | **Jarque-Bera (JB):** | 193.449 |
| **Skew:** | 0.866 | **Prob(JB):** | 9.84e-43 |
| **Kurtosis:** | 4.281 | **Cond. No.** | 3.24 |

**Table 9.** 5-Letter Identifiers Elo vs Terminal Fricative,Terminal Stop, and Initial Nasal

| | | | |
|---|---|---|---|
| **Dep. Variable:** | elo | **R-squared:** | 0.017 |
| **Model:** | OLS | **Adj. R-squared:** | 0.014 |
| **Method:** | Least Squares | **F-statistic:** | 5.872 |
| **Date:** | Wed, 29 Aug 2018 | **Prob (F-statistic):** | 0.000565 |
| **Time:** | 19:47:17 | **Log-Likelihood:** | -6187.3 |
| **No. Observations:** | 1000 | **AIC:** | 1.238e+04 |
| **Df Residuals:** | 996 | **BIC:** | 1.240e+04 |
| **Df Model:** | 3 | | |

| | **coef** | **std err** | **z** | **P> \|z\|** | **[0.025** | **0.975]** |
|---|---|---|---|---|---|---|
| **const** | 1011.6812 | 5.443 | 185.867 | 0.000 | 1001.013 | 1022.349 |
| **terminal_fricative** | 12.7265 | 9.837 | 1.294 | 0.196 | -6.554 | 32.007 |
| **terminal_stop** | 8.6200 | 8.615 | 1.001 | 0.317 | -8.265 | 25.505 |
| **initial_nasal** | 40.0655 | 10.360 | 3.867 | 0.000 | 19.760 | 60.371 |

| | | | |
|---|---|---|---|
| **Omnibus:** | 131.246 | **Durbin-Watson:** | 0.037 |
| **Prob(Omnibus):** | 0.000 | **Jarque-Bera (JB):** | 209.601 |
| **Skew:** | 0.878 | **Prob(JB):** | 3.06e-46 |
| **Kurtosis:** | 4.396 | **Cond. No.** | 3.30 |

**Table 10.** 4-Letter Identifiers Elo vs Initial Nasal

| | | | |
|---|---|---|---|
| **Dep. Variable:** | elo | **R-squared:** | 0.014 |
| **Model:** | OLS | **Adj. R-squared:** | 0.013 |
| **Method:** | Least Squares | **F-statistic:** | 13.69 |
| **Date:** | Wed, 29 Aug 2018 | **Prob (F-statistic):** | 0.000228 |
| **Time:** | 19:47:17 | **Log-Likelihood:** | -5903.3 |
| **No. Observations:** | 1000 | **AIC:** | 1.181e+04 |
| **Df Residuals:** | 998 | **BIC:** | 1.182e+04 |
| **Df Model:** | 1 | | |

| | **coef** | **std err** | **z** | **P> \|z\|** | **[0.025** | **0.975]** |
|---|---|---|---|---|---|---|
| **const** | 1007.3665 | 2.898 | 347.604 | 0.000 | 1001.686 | 1013.047 |
| **initial_nasal** | 43.3391 | 11.715 | 3.700 | 0.000 | 20.379 | 66.300 |

| | | | |
|---|---|---|---|
| **Omnibus:** | 61.410 | **Durbin-Watson:** | 0.030 |
| **Prob(Omnibus):** | 0.000 | **Jarque-Bera (JB):** | 74.083 |
| **Skew:** | 0.588 | **Prob(JB):** | 8.19e-17 |
| **Kurtosis:** | 3.627 | **Cond. No.** | 4.07 |

**Table 11.** 4-Letter Identifiers Elo vs Terminal Voiceless Consonant

| | | | |
|---|---|---|---|
| **Dep. Variable:** | elo | **R-squared:** | 0.003 |
| **Model:** | OLS | **Adj. R-squared:** | 0.002 |
| **Method:** | Least Squares | **F-statistic:** | 2.694 |
| **Date:** | Wed, 29 Aug 2018 | **Prob (F-statistic):** | 0.101 |
| **Time:** | 19:47:17 | **Log-Likelihood:** | -5909.2 |
| **No. Observations:** | 1000 | **AIC:** | 1.182e+04 |
| **Df Residuals:** | 998 | **BIC:** | 1.183e+04 |
| **Df Model:** | 1 | | |

| | **coef** | **std err** | **z** | **P> \|z\|** | **[0.025** | **0.975]** |
|---|---|---|---|---|---|---|
| **const** | 1006.6304 | 3.532 | 285.034 | 0.000 | 999.709 | 1013.552 |
| **terminal_voiceless** | 9.6553 | 5.882 | 1.641 | 0.101 | -1.874 | 21.185 |

| | | | |
|---|---|---|---|
| **Omnibus:** | 59.339 | **Durbin-Watson:** | 0.006 |
| **Prob(Omnibus):** | 0.000 | **Jarque-Bera (JB):** | 70.363 |
| **Skew:** | 0.585 | **Prob(JB):** | 5.26e-16 |
| **Kurtosis:** | 3.565 | **Cond. No.** | 2.42 |

**Table 12.** 4-Letter Identifiers Elo vs Terminal Voiced Obstruent

| | | | |
|---|---|---|---|
| **Dep. Variable:** | elo | **R-squared:** | 0.011 |
| **Model:** | OLS | **Adj. R-squared:** | 0.010 |
| **Method:** | Least Squares | **F-statistic:** | 12.26 |
| **Date:** | Wed, 29 Aug 2018 | **Prob (F-statistic):** | 0.000484 |
| **Time:** | 19:47:17 | **Log-Likelihood:** | -5905.0 |
| **No. Observations:** | 1000 | **AIC:** | 1.181e+04 |
| **Df Residuals:** | 998 | **BIC:** | 1.182e+04 |
| **Df Model:** | 1 | | |

| | **coef** | **std err** | **z** | **P> \|z\|** | **[0.025** | **0.975]** |
|---|---|---|---|---|---|---|
| **const** | 1004.3789 | 3.394 | 295.911 | 0.000 | 997.726 | 1011.031 |
| **terminal_obstruents** | 21.0314 | 6.007 | 3.501 | 0.000 | 9.257 | 32.806 |

| | | | |
|---|---|---|---|
| **Omnibus:** | 68.273 | **Durbin-Watson:** | 0.023 |
| **Prob(Omnibus):** | 0.000 | **Jarque-Bera (JB):** | 84.435 |
| **Skew:** | 0.622 | **Prob(JB):** | 4.63e-19 |
| **Kurtosis:** | 3.691 | **Cond. No.** | 2.45 |

**Table 13.** 4-Letter Identifiers Elo vs Initial Nasal and Terminal Voiceless Consonant

| | | | |
|---|---|---|---|
| **Dep. Variable:** | elo | **R-squared:** | 0.018 |
| **Model:** | OLS | **Adj. R-squared:** | 0.016 |
| **Method:** | Least Squares | **F-statistic:** | 8.767 |
| **Date:** | Wed, 29 Aug 2018 | **Prob (F-statistic):** | 0.000168 |
| **Time:** | 19:47:17 | **Log-Likelihood:** | -5901.5 |
| **No. Observations:** | 1000 | **AIC:** | 1.181e+04 |
| **Df Residuals:** | 997 | **BIC:** | 1.182e+04 |
| **Df Model:** | 2 | | |

| | **coef** | **std err** | **z** | **P> \|z\|** | **[0.025** | **0.975]** |
|---|---|---|---|---|---|---|
| **const** | 1003.1573 | 3.593 | 279.201 | 0.000 | 996.115 | 1010.199 |
| **initial_nasal** | 44.7962 | 11.742 | 3.815 | 0.000 | 21.781 | 67.811 |
| **terminal_voiceless** | 11.1807 | 5.858 | 1.909 | 0.056 | -0.301 | 22.663 |

| | | | |
|---|---|---|---|
| **Omnibus:** | 60.388 | **Durbin-Watson:** | 0.037 |
| **Prob(Omnibus):** | 0.000 | **Jarque-Bera (JB):** | 72.137 |
| **Skew:** | 0.588 | **Prob(JB):** | 2.17e-16 |
| **Kurtosis:** | 3.592 | **Cond. No.** | 4.42 |

**Table 14.** 4-Letter Identifiers Elo vs Initial Nasal,Terminal Voiceless Consonant, and Terminal Voiced Obstruent

| | | | |
|---|---|---|---|
| **Dep. Variable:** | elo | **R-squared:** | 0.038 |
| **Model:** | OLS | **Adj. R-squared:** | 0.035 |
| **Method:** | Least Squares | **F-statistic:** | 12.44 |
| **Date:** | Wed, 29 Aug 2018 | **Prob (F-statistic):** | 5.44e-08 |
| **Time:** | 19:47:17 | **Log-Likelihood:** | -5891.0 |
| **No. Observations:** | 1000 | **AIC:** | 1.179e+04 |
| **Df Residuals:** | 996 | **BIC:** | 1.181e+04 |
| **Df Model:** | 3 | | |

| | **coef** | **std err** | **z** | **P> \|z\|** | **[0.025** | **0.975]** |
|---|---|---|---|---|---|---|
| **const** | 989.1805 | 4.890 | 202.280 | 0.000 | 979.596 | 998.765 |
| **initial_nasal** | 42.8709 | 11.457 | 3.742 | 0.000 | 20.416 | 65.326 |
| **terminal_voiceless** | 25.2412 | 6.727 | 3.752 | 0.000 | 12.057 | 38.425 |
| **terminal_obstruents** | 32.3465 | 6.839 | 4.730 | 0.000 | 18.943 | 45.750 |

| | | | |
|---|---|---|---|
| **Omnibus:** | 72.454 | **Durbin-Watson:** | 0.081 |
| **Prob(Omnibus):** | 0.000 | **Jarque-Bera (JB):** | 90.873 |
| **Skew:** | 0.643 | **Prob(JB):** | 1.85e-20 |
| **Kurtosis:** | 3.726 | **Cond. No.** | 4.55 |

**Table 15.** 4-Letter Identifiers Elo vs Terminal Fricative

| | | | |
|---|---|---|---|
| **Dep. Variable:** | elo | **R-squared:** | 0.003 |
| **Model:** | OLS | **Adj. R-squared:** | 0.002 |
| **Method:** | Least Squares | **F-statistic:** | 2.770 |
| **Date:** | Wed, 29 Aug 2018 | **Prob (F-statistic):** | 0.0964 |
| **Time:** | 19:47:17 | **Log-Likelihood:** | -5909.3 |
| **No. Observations:** | 1000 | **AIC:** | 1.182e+04 |
| **Df Residuals:** | 998 | **BIC:** | 1.183e+04 |
| **Df Model:** | 1 | | |

| | **coef** | **std err** | **z** | **P> \|z\|** | **[0.025** | **0.975]** |
|---|---|---|---|---|---|---|
| **const** | 1007.9550 | 3.195 | 315.490 | 0.000 | 1001.693 | 1014.217 |
| **terminal_fricative** | 11.3121 | 6.797 | 1.664 | 0.096 | -2.010 | 24.635 |

| | | | |
|---|---|---|---|
| **Omnibus:** | 62.066 | **Durbin-Watson:** | 0.006 |
| **Prob(Omnibus):** | 0.000 | **Jarque-Bera (JB):** | 74.614 |
| **Skew:** | 0.596 | **Prob(JB):** | 6.28e-17 |
| **Kurtosis:** | 3.609 | **Cond. No.** | 2.63 |

**Table 16.** 4-Letter Identifiers Elo vs Terminal Stop

| | | | |
|---|---|---|---|
| **Dep. Variable:** | elo | **R-squared:** | 0.013 |
| **Model:** | OLS | **Adj. R-squared:** | 0.012 |
| **Method:** | Least Squares | **F-statistic:** | 13.31 |
| **Date:** | Wed, 29 Aug 2018 | **Prob (F-statistic):** | 0.000278 |
| **Time:** | 19:47:17 | **Log-Likelihood:** | -5904.1 |
| **No. Observations:** | 1000 | **AIC:** | 1.181e+04 |
| **Df Residuals:** | 998 | **BIC:** | 1.182e+04 |
| **Df Model:** | 1 | | |

| | **coef** | **std err** | **z** | **P> \|z\|** | **[0.025** | **0.975]** |
|---|---|---|---|---|---|---|
| **const** | 1003.6893 | 3.363 | 298.432 | 0.000 | 997.097 | 1010.281 |
| **terminal_stop** | 22.3171 | 6.117 | 3.648 | 0.000 | 10.328 | 34.307 |

| | | | |
|---|---|---|---|
| **Omnibus:** | 63.532 | **Durbin-Watson:** | 0.028 |
| **Prob(Omnibus):** | 0.000 | **Jarque-Bera (JB):** | 76.680 |
| **Skew:** | 0.604 | **Prob(JB):** | 2.23e-17 |
| **Kurtosis:** | 3.617 | **Cond. No.** | 2.43 |

**Table 17.** 4-Letter Identifiers Elo vs Terminal Fricative and Terminal Stop

| | | | |
|---|---|---|---|
| **Dep. Variable:** | elo | **R-squared:** | 0.021 |
| **Model:** | OLS | **Adj. R-squared:** | 0.019 |
| **Method:** | Least Squares | **F-statistic:** | 10.74 |
| **Date:** | Wed, 29 Aug 2018 | **Prob (F-statistic):** | 2.44e-05 |
| **Time:** | 19:47:17 | **Log-Likelihood:** | -5899.8 |
| **No. Observations:** | 1000 | **AIC:** | 1.181e+04 |
| **Df Residuals:** | 997 | **BIC:** | 1.182e+04 |
| **Df Model:** | 2 | | |

| | **coef** | **std err** | **z** | **P> \|z\|** | **[0.025** | **0.975]** |
|---|---|---|---|---|---|---|
| **const** | 997.6954 | 4.021 | 248.151 | 0.000 | 989.815 | 1005.575 |
| **terminal_fricative** | 21.5718 | 7.222 | 2.987 | 0.003 | 7.417 | 35.727 |
| **terminal_stop** | 28.3109 | 6.502 | 4.354 | 0.000 | 15.568 | 41.054 |

| | | | |
|---|---|---|---|
| **Omnibus:** | 68.137 | **Durbin-Watson:** | 0.046 |
| **Prob(Omnibus):** | 0.000 | **Jarque-Bera (JB):** | 83.539 |
| **Skew:** | 0.627 | **Prob(JB):** | 7.24e-19 |
| **Kurtosis:** | 3.657 | **Cond. No.** | 3.26 |

**Table 18.** 4-Letter Identifiers Elo vs Terminal Fricative,Terminal Stop, and Initial Nasal

| | | | |
|---|---|---|---|
| **Dep. Variable:** | elo | **R-squared:** | 0.036 |
| **Model:** | OLS | **Adj. R-squared:** | 0.033 |
| **Method:** | Least Squares | **F-statistic:** | 11.80 |
| **Date:** | Wed, 29 Aug 2018 | **Prob (F-statistic):** | 1.35e-07 |
| **Time:** | 19:47:17 | **Log-Likelihood:** | -5892.2 |
| **No. Observations:** | 1000 | **AIC:** | 1.179e+04 |
| **Df Residuals:** | 996 | **BIC:** | 1.181e+04 |
| **Df Model:** | 3 | | |

| | **coef** | **std err** | **z** | **P> \|z\|** | **[0.025** | **0.975]** |
|---|---|---|---|---|---|---|
| **const** | 994.6756 | 4.073 | 244.231 | 0.000 | 986.693 | 1002.658 |
| **terminal_fricative** | 21.6764 | 7.167 | 3.025 | 0.002 | 7.630 | 35.723 |
| **terminal_stop** | 28.7500 | 6.468 | 4.445 | 0.000 | 16.073 | 41.427 |
| **initial_nasal** | 44.1755 | 11.624 | 3.800 | 0.000 | 21.393 | 66.958 |

| | | | |
|---|---|---|---|
| **Omnibus:** | 69.990 | **Durbin-Watson:** | 0.077 |
| **Prob(Omnibus):** | 0.000 | **Jarque-Bera (JB):** | 86.930 |
| **Skew:** | 0.632 | **Prob(JB):** | 1.33e-19 |
| **Kurtosis:** | 3.700 | **Cond. No.** | 4.35 |
