# Supplementary material for: CurmElo: The theory and practice of a forced-choice approach to producing preference rankings
Source: PLoS One. 2021 May 27;16(5):e0252145. doi: 10.1371/journal.pone.0252145 (PMC8158949; doi:10.1371/journal.pone.0252145)
Supplement: S2 Appendix — Regressions for phonological constructions on polarization. (PDF) [file pone.0252145.s002.pdf]

S2 Appendix: Polarization Regression Models. **Regressions for Phonological Constructions on Polarization.**

Table 1. 5-Letter Identifiers Polarization vs Initial Nasal and Terminal Voiceless Consonant

**Dep. Variable:**	rank_breaks	**R-squared:**	0.002
**Model:**	OLS	**Adj. R-squared:**	0.000
**Method:**	Least Squares	**F-statistic:**	1.283
**Date:**	Wed, 29 Aug 2018	**Prob (F-statistic):**	0.278
**Time:**	22:26:53	**Log-Likelihood:**	-1859.6
**No. Observations:**	1000	**AIC:**	3725.
**Df Residuals:**	997	**BIC:**	3740.
**Df Model:**	2		

	**coef**	**std err**	**z**	**P> |z|**	**[0.025**	**0.975]**
**const**	2.4947	0.068	36.550	0.000	2.361	2.628
**initial_nasal**	-0.2034	0.129	-1.578	0.115	-0.456	0.049
**terminal_voiceless**	-0.0231	0.100	-0.232	0.817	-0.218	0.172

**Omnibus:**	83.175	**Durbin-Watson:**	2.034
**Prob(Omnibus):**	0.000	**Jarque-Bera (JB):**	105.922
**Skew:**	0.709	**Prob(JB):**	9.99e-24
**Kurtosis:**	3.730	**Cond. No.**	3.07

Table 2. 5-Letter Identifiers Polarization vs Initial Nasal,Terminal Voiceless Consonant, and Terminal Voiced Obstruent

**Dep. Variable:**	rank_breaks	**R-squared:**	0.002
**Model:**	OLS	**Adj. R-squared:**	-0.001
**Method:**	Least Squares	**F-statistic:**	0.8567
**Date:**	Wed, 29 Aug 2018	**Prob (F-statistic):**	0.463
**Time:**	22:26:53	**Log-Likelihood:**	-1859.6
**No. Observations:**	1000	**AIC:**	3727.
**Df Residuals:**	996	**BIC:**	3747.
**Df Model:**	3		

	**coef**	**std err**	**z**	**P> |z|**	**[0.025**	**0.975]**
**const**	2.4963	0.084	29.599	0.000	2.331	2.662
**initial_nasal**	-0.2034	0.129	-1.576	0.115	-0.456	0.049
**terminal_voiceless**	-0.0247	0.112	-0.221	0.825	-0.244	0.194
**terminal_obstruents**	-0.0044	0.134	-0.033	0.974	-0.267	0.258

**Omnibus:**	83.181	**Durbin-Watson:**	2.034
**Prob(Omnibus):**	0.000	**Jarque-Bera (JB):**	105.940
**Skew:**	0.709	**Prob(JB):**	9.89e-24
**Kurtosis:**	3.730	**Cond. No.**	3.67

Table 3. 5-Letter Identifiers Polarization vs Terminal Fricative, Terminal Stop, and Initial Nasal

**Dep. Variable:**	rank_breaks	**R-squared:**	0.007
**Model:**	OLS	**Adj. R-squared:**	0.004
**Method:**	Least Squares	**F-statistic:**	2.034
**Date:**	Wed, 29 Aug 2018	**Prob (F-statistic):**	0.107
**Time:**	22:26:53	**Log-Likelihood:**	-1857.5
**No. Observations:**	1000	**AIC:**	3723.
**Df Residuals:**	996	**BIC:**	3743.
**Df Model:**	3		

	**coef**	**std err**	**z**	**P> |z|**	**[0.025**	**0.975]**
**const**	2.4497	0.071	34.569	0.000	2.311	2.589
**terminal_fricative**	0.2501	0.140	1.784	0.074	-0.025	0.525
**terminal_stop**	-0.0348	0.111	-0.314	0.753	-0.252	0.183
**initial_nasal**	-0.2019	0.129	-1.566	0.117	-0.455	0.051

**Omnibus:**	81.069	**Durbin-Watson:**	2.040
**Prob(Omnibus):**	0.000	**Jarque-Bera (JB):**	102.251
**Skew:**	0.701	**Prob(JB):**	6.26e-23
**Kurtosis:**	3.699	**Cond. No.**	3.30

Table 4. 4-Letter Identifiers Polarization vs Initial Nasal and Terminal Voiceless Consonant

**Dep. Variable:**	rank_breaks	**R-squared:**	0.005
**Model:**	OLS	**Adj. R-squared:**	0.003
**Method:**	Least Squares	**F-statistic:**	2.715
**Date:**	Wed, 29 Aug 2018	**Prob (F-statistic):**	0.0667
**Time:**	22:26:53	**Log-Likelihood:**	-1910.7
**No. Observations:**	1000	**AIC:**	3827.
**Df Residuals:**	997	**BIC:**	3842.
**Df Model:**	2		

	**coef**	**std err**	**z**	**P> |z|**	**[0.025**	**0.975]**
**const**	2.7255	0.065	42.232	0.000	2.599	2.852
**initial_nasal**	-0.3777	0.201	-1.881	0.060	-0.771	0.016
**terminal_voiceless**	0.1496	0.110	1.360	0.174	-0.066	0.365

**Omnibus:**	42.601	**Durbin-Watson:**	1.915
**Prob(Omnibus):**	0.000	**Jarque-Bera (JB):**	47.276
**Skew:**	0.532	**Prob(JB):**	5.42e-11
**Kurtosis:**	3.053	**Cond. No.**	4.42

Table 5. 4-Letter Identifiers Polarization vs Initial Nasal,Terminal Voiceless Consonant, and Terminal Voiced Obstruent

**Dep. Variable:**	rank_breaks	**R-squared:**	0.006
**Model:**	OLS	**Adj. R-squared:**	0.003
**Method:**	Least Squares	**F-statistic:**	2.053
**Date:**	Wed, 29 Aug 2018	**Prob (F-statistic):**	0.105
**Time:**	22:26:53	**Log-Likelihood:**	-1910.4
**No. Observations:**	1000	**AIC:**	3829.
**Df Residuals:**	996	**BIC:**	3848.
**Df Model:**	3		

	**coef**	**std err**	**z**	**P> |z|**	**[0.025**	**0.975]**
**const**	2.6859	0.086	31.338	0.000	2.518	2.854
**initial_nasal**	-0.3832	0.201	-1.907	0.056	-0.777	0.011
**terminal_voiceless**	0.1894	0.124	1.533	0.125	-0.053	0.432
**terminal_obstruents**	0.0915	0.127	0.723	0.470	-0.157	0.340

**Omnibus:**	42.704	**Durbin-Watson:**	1.914
**Prob(Omnibus):**	0.000	**Jarque-Bera (JB):**	47.399
**Skew:**	0.533	**Prob(JB):**	5.10e-11
**Kurtosis:**	3.055	**Cond. No.**	4.55

Table 6. 4-Letter Identifiers Polarization vs Terminal Fricative, Terminal Stop, and Initial Nasal

**Dep. Variable:**	rank_breaks	**R-squared:**	0.005
**Model:**	OLS	**Adj. R-squared:**	0.002
**Method:**	Least Squares	**F-statistic:**	1.955
**Date:**	Wed, 29 Aug 2018	**Prob (F-statistic):**	0.119
**Time:**	22:26:53	**Log-Likelihood:**	-1910.7
**No. Observations:**	1000	**AIC:**	3829.
**Df Residuals:**	996	**BIC:**	3849.
**Df Model:**	3		

	**coef**	**std err**	**z**	**P> |z|**	**[0.025**	**0.975]**
**const**	2.7241	0.074	36.706	0.000	2.579	2.870
**terminal_fricative**	0.0480	0.136	0.354	0.724	-0.218	0.314
**terminal_stop**	0.1648	0.122	1.351	0.177	-0.074	0.404
**initial_nasal**	-0.3922	0.202	-1.939	0.053	-0.789	0.004

**Omnibus:**	43.935	**Durbin-Watson:**	1.911
**Prob(Omnibus):**	0.000	**Jarque-Bera (JB):**	48.905
**Skew:**	0.541	**Prob(JB):**	2.40e-11
**Kurtosis:**	3.067	**Cond. No.**	4.35
