# Supplementary material for: CurmElo: The theory and practice of a forced-choice approach to producing preference rankings
Source: PLoS One. 2021 May 27;16(5):e0252145. doi: 10.1371/journal.pone.0252145 (PMC8158949; doi:10.1371/journal.pone.0252145)
Supplement: S3 Appendix — (PDF) [file pone.0252145.s003.pdf]

S3 Appendix: Robustness Checks. We conducted a number of robustness checks to determine how sensitive the rankings and Elo values are to various parameter permutations and subsamples. We provide two types of metrics to evaluate these robustness tests: vector metrics and quantile metrics. For the vector metrics we calculated the Euclidean distance and the Cosine distance between each parameter permutation / subsample and the elo rankings used in the main result. To ensure the rankings were compatible all rankings were sorted before Euclidean distance and Cosine distance were calculated. The quantile rankings measure for how many words changed quantile from the main ranking to the new parameterizations/ subsample. Total Quantile Changes measure the total number of words which change quantiles. Large Quantile Changes measures the number of words that change by more than one quantile across rankings. For example if a word moved from the 1st quantile to the 3rd quantile that would be a large quantile change while moving from the 4th quantile to the 3rd quantile would not be a large quantile change. Table 1 shows the results of robustness tests involving changing the parameters K, R_D, and R_0 for the 4-Letter Words. Table 2 shows the results of robustness tests involving changing the parameters K, R_D, and R_0 for the 5-Letter Words. Changing the parameter R_0 did not result in any changes in quantiles suggesting that the rankings are quite robust to changes in this paramter. This is to be expected since this parameter effectively sets the mean of the distribution. For the parameters K and R_D there was some significant changes across quantiles but relatively few large quantile changes. Combining this results with the high Euclidean distances and low Cosine distances suggests that when there is movement in the rankings it tends to be either within quantiles or to neighboring quantiles. This suggests that the strengths being represented are reasonably robust for the purposes of analysis at the quantile level.

Table 1. Impact of Altering Elo Parameters K, R_D, and R_0 for 4-Letter Words

	Euclidean Distance	Cosine Distance	Total Quantile Changes	Large Quantile Changes
K=10,R_D=400,R_0 =1000	1462.1354023297765	0.000996233972913485	66	0
K=20,R_D=40,R_0 =1000	1488.379835355675	0.0007983886983470301	397	35
K=20,R_D=200,R_0 =1000	458.01490010605215	$7.3278287038403 * 10^{-5}$	116	0
K=20,R_D=800,R_0 =1000	299.9538036737356	$3.2074441283502786 * 10^{-5}$	66	0
K=40,R_D=400,R_0 =1000	3173.3343683362286	0.004067749524301556	116	0
K=100,R_D=400,R_0 =1000	13568.741873752411	0.05279736768457266	301	5
K=20,R_D=400,R_0 =500	15811.388300838196	0.0036169331038051755	0	0
K=20,R_D=400,R_0 =1500	15811.388300840568	0.00042327336810310534	0	0

Table 2. Impact of Altering Elo Parameters K, R_D, and R_0 for 5-Letter Words

	Euclidean Distance	Cosine Distance	Total Quantile Changes	Large Quantile Changes
K=10,R_D=400,R_0 =1000	2013.4516735243474	0.0017235465185065912	68	0
K=20,R_D=40,R_0 =1000	1849.5294910669072	0.0008902119966440747	358	23
K=20,R_D=200,R_0 =1000	669.4446173989384	0.0001078847431832397	104	0
K=20,R_D=800,R_0 =1000	513.832153618179	$7.061834462374073 * 10^{-5}$	68	0
K=40,R_D=400,R_0 =1000	4434.730032560456	0.006349615408436593	104	0
K=100,R_D=400,R_0 =1000	18601.25411032846	0.06509600612691702	258	8
K=20,R_D=400,R_0 =500	15811.388300842149	0.005802629738783094	0	0
K=20,R_D=400,R_0 =1500	15811.388300841834	0.0007130073184216101	0	0

We also did subsample analysis on the 4-Letter words by analyzing a certain fraction of the comparisons per person. Due to technical restrictions of the version of the CuremElo software used to to collect the data for the 5-Letter words this type of analysis is not possible for the 5-Letter words. The first type of analysis we did was burn in burn out analysis, where we drop the first and last n comparisons. We conducted this analysis with n=5 and n=10, resulting in samples of size 40 and 30 per person. To determine whether there was anything unique about the first and last comparisons (warmup period, or exhasution) we also analyzed subsamples with 30 and 40 randomly selected comparisons per person. Finally to test whether the structure of the words might might be inducing non independence across comparisons, we looked at only comparisons where none of the words share any consonants with the previous comparison. The results of these tests are show in Table 3. These subsamples have significantly more total quantile changes as well as large quantile changes. It is important to note that these tests involve dropping large amounts of the sample, so it is not entirely surprising that this could lead to significant changes. Additionally the numbers of quantile changes are similar between burning the first and last 5 and randomly sampling 40 comparison and the quantile changes for burning the first and last 10 comparions is also similiar to randomly sampling 30 comparisons per person. This suggests that a significant fraction of this difference is due to dropping the data and is not necessarily evidence of non independence across comparisons. These results are still somewhat consistent with non independence across comparisons but it clear from these tests.

Table 3. Impact of Data Dropping for 4-Letter Words

	Euclidean Distance	Cosine Distance	Total Quantile Changes	Large Quantile Changes
Burn 5	1117.7004194218039	0.0005986894977070278	352	30
Burn 10	1657.689512724917	0.0013071351486236837	487	91
No Serial Consonants	1974.4592927130159	0.0018527159833675588	547	159
Random 30	1520.9221634223636	0.0010994159028988593	485	92
Random 40	993.507855335927	0.0004723665457366799	318	18
