# Supplementary material for: CurmElo: The theory and practice of a forced-choice approach to producing preference rankings
Source: PLoS One. 2021 May 27;16(5):e0252145. doi: 10.1371/journal.pone.0252145 (PMC8158949; doi:10.1371/journal.pone.0252145)
Supplement: S4 Appendix — This table contains the complete list of identifiers used. (PDF) [file pone.0252145.s004.pdf]

S4 Appendix: List of Identifiers. **This table contains the complete list of identifiers used.**

4-Letter	5-Letter
ivek	pesam
asiq	jayor
avox	kewos
ejon	mavey
omog	coyam
otif	fexen
ojav	pebel
ebud	jupar
ufix	cosop
anix	lozir
awek	koduk
epek	kovaq
izus	mazob
owek	camaz
ozis	bumak
azop	kujol
ixel	falom
emev	poqit
opaw	gosaw
uzus	hosop
uzok	madik
ovay	jutah
axoy	naroc
ozir	lenim
imeg	lelip
atux	gomos
otiz	nubul
uvap	paqel
awax	nuvis
izop	kozec
okoy	dajav
uboz	hamok
ebak	luwog
icoy	korav
enud	puzat
omaj	coxar
oyek	kehab
ebek	gupac
ibuy	hugum
ezip	nalaj
ovox	jaxam
eviv	nejix
ewop	kayus
equm	helad
ezub	nawam
opaz	neroy
enux	maviz
ikab	nipiq
ewoq	cebis

axah	gixan
emuv	mibob
osaz	banom
okap	lilap
ubob	nadog
evuz	pizaz
uvaq	pukap
oxef	deneh
eluz	melox
ufam	nowan
uweg	pefay
ovaq	hazud
enub	finoy
oliq	qavan
oyix	cazup
uzab	mezed
emoc	carir
ewiz	moruz
ecox	kisop
oxoh	mideh
umeg	bavez
etug	peber
oxim	jomic
abux	kojeb
ageg	dapup
ojay	midux
ayom	koyev
ewut	laral
ipox	luxos
ehoc	cupun
ugot	lobud
ezej	neliz
umaj	luzaf
agud	hawoh
udur	kogas
azuc	jusol
omeb	mefer
ecup	mavaz
eqiv	maqet
uwan	livud
izip	mumek
ezot	pixed
azod	pagol
usiv	jatet
awup	dacoy
uzim	jalav
ovax	fozer
opeg	leyah
upad	casuk
oxaz	hidar
obeq	mikaw
ezac	demox

ukiz	hinaj
oniq	ceyos
esoy	mofex
ebaj	gozik
ayug	maqis
ihub	bajoc
umaw	kimoz
uqiz	jezed
afav	jufah
owat	jolot
odiz	mifun
udog	humeg
uxal	miroj
uraj	panuf
olab	cunim
uwos	naroy
odaj	fazos
edav	kivam
oqal	lezul
emix	meqac
atez	pedax
adug	gupil
aqan	lohaw
ogix	jareh
ataj	povip
afab	gudem
adez	deyal
exaz	mosiv
urib	micax
esug	luqem
ojep	culah
owok	panoc
ufom	jawok
inaz	jaluk
obuk	gidem
adoy	pimac
umix	gemey
ovix	fazov
onoq	kuzac
eyab	koriv
uvir	defos
icaw	bokac
iwid	cufay
evev	luciy
odip	bageg
oqim	fazip
ozec	dafir
iwal	levid
ezif	nizaz
ucaw	kehid
ehum	diriv
ejav	namuc

esok	mogip
axog	mekeb
omeq	mepoh
imeq	nayim
olaq	kusit
oteq	jecid
eraw	bomav
umez	nirab
upez	hurod
oqos	mutiz
oket	hunaq
aqic	meyir
osuk	cudip
aqom	qador
ozit	korit
azij	cinok
ohep	kuyem
aqed	maheg
azub	pogok
avaw	hodev
ojeb	koyem
izax	kenux
odab	muvoc
idoz	famoy
ofaj	pemeg
aqac	duzin
izig	mivon
ojaw	muvit
aduw	gulac
ucog	hikop
esuz	miloc
urix	legom
axeb	davuk
atix	dedow
ezeb	pujom
uzid	pocor
orob	fabox
opaq	jigas
uzif	lulaq
ojad	pizah
avux	gawus
ubaz	ganur
uqil	gumad
atev	pufun
owuk	foraj
owol	kadul
ozaw	muluq
emoz	lifix
uzev	cofic
ajun	hukar
ocuz	jahaw
apaj	mojaw

usay	bejon
odid	hisaf
ojex	necuz
azuy	payom
esiz	gumon
ameq	decek
ejox	kebad
ebop	kulem
okif	nuxos
ijun	geroz
oyok	jixil
uleq	gemud
uxir	diroc
ewun	mojic
icaj	kuwis
axez	neyus
ocaz	jurib
atug	jusev
ezog	mafum
ubix	jajoy
oyak	bulem
arux	bapas
oqey	jexic
areq	mafur
opup	laxip
esog	lamoz
oxux	natap
onip	kefoz
aqaq	gunek
ozat	kugos
oqon	qetas
udiz	pudew
ukul	kegit
agug	namot
exoc	juzil
axuz	bemaf
aqij	finij
ovus	negaz
uxer	niwal
egev	kedew
enex	canof
ihol	fanap
ayaf	hopaf
uxix	lugof
ohiv	lejag
osot	cadej
acax	melaq
ozej	golug
aleq	bacoy
izik	hixit
ehuz	bodof
uruq	nidac

igak	jukug
utiq	kopip
eqob	gaqon
azup	cuzoy
ujok	hadof
ataw	cecig
ijex	maded
ezoh	hikas
uxim	laqiz
ozov	mumaf
iqex	jufat
ejul	jahev
ezup	foxuy
ebaf	butaz
eqon	locun
ajag	muhaz
ujen	nofen
efob	pamaq
eqot	lipob
uqad	mopot
acag	baziq
osih	lecuh
ozaj	jedap
usex	nisev
uqal	doqor
ejom	fawun
ulot	busix
abej	bizej
unuv	bamuc
okof	mufur
ulob	nofiz
izot	bozit
ogaf	bobeh
ogaw	cusud
obuf	buseq
apey	boliy
ohuz	hagac
esif	dihet
osoy	lajak
isuc	cepoz
opef	miseh
ateg	luyak
aqad	janoz
ibud	fabix
afep	jelag
apew	pakih
oxap	pevid
emiz	nanic
idug	babap
uboy	hocun
omob	forek
ubop	nugeb

ogem	bonij
obeg	fehaz
esul	husig
ujaj	mexoh
izec	galuv
imaw	fajul
anuz	payex
oxat	fanuv
evaw	jupel
imom	camux
izep	mabub
enib	dodeg
odoh	qehad
oxex	firup
ipeq	cusem
ejim	japaf
ojug	bewej
ekit	lokip
azeh	bizuq
ilox	nejux
oxeb	gufas
oyey	luveh
efux	fahah
ajav	bafen
oyoj	butaf
ezex	lidag
ozob	dimal
egif	qovel
ekux	domav
iduh	bajuw
ejiv	bexod
eqoc	gilox
opuf	copox
epof	lisiy
ucaz	dutos
usuk	loqen
emuj	jewem
egur	pomew
isuv	hiwer
iwek	lapaq
imif	kisid
ewof	dawos
akiy	dowir
owef	harok
ejem	qesex
eneg	ciriv
ajof	kenuy
obiz	jiwin
avew	pibab
atey	lofow
ikox	lufim
osob	faroq

oqaz	dawom
ivip	hikip
eruz	jekoq
exeb	qapor
uvub	nobuq
akeb	hayup
efay	pucax
iwiz	lijat
icev	bamam
uqox	gigig
izuj	bavim
ubot	godoh
ureh	lofic
ekaf	neqet
oqet	kujih
owik	fekey
edup	dizus
ojil	dimuz
epum	cetef
upuq	juwac
aseg	cimoz
uqaw	jahuh
uzub	mibom
ixoy	kagat
izoc	bijek
upaj	gakaz
efaq	ducoz
iwof	kuroc
avuk	bocut
umuy	maxih
utiv	fapod
ecuf	manup
ivuz	kuzaj
igab	focom
efib	hehum
ozuz	jiroy
edih	gupeq
idix	neyat
obef	jucin
iwoj	husaw
ipob	nalew
asiv	qinik
ekat	qenon
ihak	buzaz
axak	bibil
owet	gemeh
ohax	jizof
izex	jeqon
amup	fuxum
exuh	qukum
ehaf	kajip
uhip	qimix

ekik	biwuk
uzuf	hokuj
ubox	muhaf
usuq	fijot
awur	dayip
owug	hahek
ojon	jirem
ofok	mituh
ivaw	hucaw
ukoz	gitab
ubup	nigis
ibek	hizid
uziz	moruv
ezuv	boveq
opiw	qayuk
ajif	kafux
ogab	qotey
udub	micok
ixeb	lepey
exoz	nibat
ucah	bilaq
umav	dijeh
uwab	bisup
ekew	furub
uyox	japoz
ufub	deqeb
ehax	dukup
ixak	heyoz
oyis	gipum
uvim	gavuc
odod	bucom
onuj	capeb
owud	kazuj
ozix	nupub
otaj	jekox
ayuf	kawir
ujur	noyat
ajeb	niqin
aniw	cuxur
asuh	dijun
ibup	bumuy
ucib	bopad
ezuw	nuyak
uteb	kofup
evaj	mifep
uwec	ponum
ehix	gewom
iboq	lovuj
ojix	qaric
efif	kakox
ocav	cotoj
utaj	jowog

okut	dobot
ihoz	kipoh
axaq	bakux
ocox	bumec
omoq	nelit
eqin	kuqoc
otof	mibad
uxas	febaq
oxop	qacam
orud	fojub
uhim	gapib
aqux	giqar
uyac	pelub
uwap	jinop
asiw	lafec
ogoc	laxub
oqeb	cicum
efok	dosoz
ojel	bihir
ekok	mugeh
unih	fizeb
exuk	qamuh
uloj	bohud
okuc	kexuw
ufuy	lulew
awaf	foyar
ikak	bipam
ehof	hihug
oqan	maxaq
ipuz	nifum
iwak	fugum
icud	notak
ebup	mehor
avep	gujad
uboc	loxab
ifeg	biyom
ufug	maxoj
eteb	fojix
eguk	qupep
ofov	fihar
obeb	bofef
owev	dodas
uvut	padeb
avuv	gigox
izaj	bumaf
ohiz	fipev
ejij	jehas
eqis	gokuv
eyix	jujuc
iwuq	mudut
uluv	deguv
upuv	hidep

ubag	loxoz
ecow	cudol
odij	qozus
osib	dibed
eqec	domif
etew	ceriy
oqur	muxil
awut	kosob
osah	pelif
odiq	fariw
ohet	qaxol
ituk	lujaf
uhoc	piqez
upip	diwab
uxaq	koxus
ewit	lanoq
uwur	dabaf
ocig	buduf
ocix	jutur
okog	bexoh
uvaj	gafux
ejap	jiyag
epab	fazuf
emob	keleq
ufez	qameh
uvow	japum
ahiq	hexaq
ujes	cibam
ewav	kesuv
ukuy	cokud
omeh	dekip
udej	perap
ekak	hitej
onaf	pisof
otih	hedul
ogep	nuvok
afoq	biwar
ewad	hivoy
osuz	liper
ihib	nunif
iqem	qirab
uhod	mabod
ofeb	govez
utav	kekap
omih	giniq
utif	haceh
eqol	jafaf
esud	nolur
ayuq	buqov
oyur	burug
uwit	fahij
owep	gonuc

oyic	qomap
onuv	nagiq
ekuh	fapif
aziy	koguf
aheh	kixan
ujip	qobeh
iqom	cukep
uqat	gafig
uluh	qujet
ahuc	hidiz
utib	kamip
aliw	pewom
acuh	dafum
uveq	cuzul
ezaw	dahow
upuj	pexih
ipej	joyuk
ogaj	diqom
ofib	niniq
ewik	meyiy
enag	jaroj
idux	pecoq
inuj	cucuk
iqew	gerev
ezuc	becef
utof	boruy
owav	nefag
apuq	nicoq
unub	facuz
ewuf	jidit
ayuy	jekiy
isiz	fasij
ofuk	fukoh
iyit	kunag
igoc	kacex
ajuy	qemor
ifaf	pivun
ivoh	nomep
okiv	dilux
ecej	cecay
ihaq	biseq
iyeh	bowal
uvud	dedik
ukec	luhid
atuy	qodeb
utog	kimof
akof	nogex
imuh	jataj
ovug	fisok
ehoq	qawep
ucux	badod
ucax	kujod

ikum	fahuk
ekoz	jojuy
akeg	gudik
ovib	foxih
uzuq	gewus
uhux	hetaq
apuv	nakix
ihig	leqop
uyov	juyox
eviz	lihih
abuq	lojut
azuw	mohiw
ojoz	cebuq
uvek	dejiw
ifiz	qoroc
egeb	nipoz
ojuh	baxud
ixid	cukip
ucuf	lagoc
iqed	kigog
ekid	joxiy
equx	jucev
ugos	qemak
ujoz	lezax
aduv	besow
uxuy	guyep
ikuz	hozej
ecur	qecim
uyip	decix
ucod	gosac
eyep	juwem
ewuh	gegiv
owid	bufav
upuz	dihep
ajep	ciqog
iloq	qiruc
ajiv	nuzot
inup	didiv
owig	dofuc
ufit	lowop
atuj	hifan
ocof	fiteb
uboq	dolih
ucuw	bedif
edeg	ciziy
ogeb	baqey
ibef	naqup
iwum	linuw
uyut	dokek
oqek	fujed
enaw	cukol
ogog	fizim

edaq	fopiv
eziy	qehab
uzeb	jecir
icag	bijag
edaj	lojed
ahoz	dogoc
ogoq	febum
igah	fafuf
afuz	pikaq
uweq	nojiy
eqez	bodid
iwef	fizuz
iqef	midut
egux	bequv
ovof	nibod
axuj	cunoj
owom	guluw
ulim	dadef
ucay	mumop
ujir	pehem
uwal	kecic
uzuw	qucir
aqov	coyul
ugic	lohuh
icuv	heqij
izux	gapif
uvoq	kiyir
atuh	leqem
oxud	jukig
ixow	fovif
ozuy	qegoy
ekoc	deruq
avaj	huhuk
acew	pahif
eduw	nuxok
umew	bomip
ivez	fuyoh
ecuh	nonoj
osij	jaqew
ixaf	qegem
ufeb	hoyil
asoz	mugiy
ohuj	fijiv
ikof	gisif
efiq	nuhuz
uvej	bihib
ugeg	jagij
eboj	mufuf
ixal	pojik
oqib	lacuc
onoj	hodud
obup	bobod

ewip	humiy
ujus	hodog
ihel	nanuw
ukuk	papiq
usux	mipef
uyux	pezuh
ijoq	qayiv
eqes	dudoq
ufoh	mukiy
ayow	kogiy
ulej	qepar
ecaf	jajix
uvab	jojaq
equh	cagiq
ihuw	kugux
ayiw	hefom
iwac	qamom
ejeb	qonip
iduy	jemej
epuk	giviq
ujim	jociy
uhiz	qibog
ekoq	navep
ehog	pojuv
ohub	gupok
ukaq	joviw
abup	geluq
ofec	fevut
iwip	mijiz
iwiq	mapej
uhuv	pifil
ehuq	lawuv
eyir	luwub
opih	gedij
ugor	duyit
ofuq	husit
idej	newew
uxop	juwos
ugiz	nijef
iwuc	heham
ipib	gimaw
ixac	hufuh
itaw	cikoz
ehoh	dewil
uxam	neriy
uvav	qeguz
uyeh	kugog
oguw	qugac
iqoz	goqaj
uzof	kukij
uxac	moqac
evuq	qexix

axuc	geciv
uqef	qapem
oqut	juzud
uyif	qimec
omuy	jofoc
unaj	jebut
uniz	pituf
aguf	pobuz
ikuk	ququl
oqez	pomiy
oqul	pewep
uyem	nuguk
uwil	fisof
aqiy	jufad
iwuy	dufic
ibip	dajuf
uwaw	neqeq
ahek	qucic
uyis	jufej
utej	muxuk
uqiq	bigaj
uzug	mikox
opoq	kazij
icak	baziy
ipul	femof
uqiw	keqif
ejev	jekeh
ujux	qebux
epuy	gibep
umub	fapih
ufob	peqom
ebip	bewef
ituw	qecut
uhih	kixex
ugiv	fuqeh
ojip	gasuq
otoj	gosod
uneb	qewaj
uyey	gicuk
iduw	jaqej
ewiv	lejij
ekiy	codej
uvux	kocuj
efoh	cunuh
udud	baqoc
egay	cigup
udoj	kuhix
unuy	huqiv
icuh	pewuk
uroj	gahoz
upuf	duzug
ejep	fimup

ageq	qeyaw
ixod	qofis
iyut	jaxug
oxak	gitif
ayeq	bapef
icih	futuj
ofih	juyed
ezeg	gileq
ubij	miguw
igex	boqon
uyaw	capeh
ewiq	gixub
obuj	meyiq
uneh	geqor
ovuq	pucoc
ozoq	pahih
oqak	qiruq
iviq	cecec
uriy	hepoj
ojux	qoneb
imuw	lunij
igaq	jepiv
ofoc	qawaj
oyiv	qepov
iyej	qimuz
iwom	kigay
upiw	biqix
esuw	qupov
ijol	huvib
ujud	cemif
awih	nagip
ugeh	dedav
uyeb	quhec
ugaq	nuhux
aqey	bapup
uzaj	fuqug
ojig	nacih
ulij	qidew
afeg	jucuj
ukib	difiv
uqex	boguw
uxut	goqeg
uziy	hociy
oqiw	ciyip
uxaw	bowiv
oxuj	fufob
ipuj	gevip
iwet	hajoj
otuj	hofoc
ihif	nekuc
equq	qicot
awus	qexay

upej	furep
iqen	ceqem
adih	fihoq
aqaj	qihux
owiv	muyoy
oqok	foviz
ugej	qaqac
iruq	jujif
eyoj	qekah
ixud	qamoh
egew	jixek
aqeg	noduq
ahef	cicaj
obik	kuxaj
uvep	qoyin
ucuv	qoqaw
iyag	mihid
afoj	guyaj
uraw	ligeg
ufiv	norur
uqeg	lereg
ucoh	qadug
ohix	nosif
iyev	fugiq
ujiv	giheb
uyul	gowoc
oqic	guqur
uwuz	qediy
ujif	jucew
ixub	gugej
oxoq	cewoh
ugez	guxun
ugih	qevoj
ugeq	desaq
iquw	qafun
eqoj	jiyuy
uqoj	cefuc
ocij	guheh
iwev	gexud
ubuq	jijob
ugop	ligib
ugaj	giqir
ihog	nujuj
awoj	fukik
ifeh	gosux
iyiq	fideh
iyif	hiziq
igub	hehil
uyez	gusuz
uxev	fodeh
ihiy	nuzoq
iqug	kogiw

ukiw	qejev
uyeq	jilih
ukij	hihuh
ujaq	qexag
ejiw	nutuj
uguf	fakag
uwiq	qanej
ipuw	qezuv
igew	peqef
ijaj	pigef
ubih	noxoj
oqof	qagiw
idiy	qoqiq
ikux	liliw
ifof	nafug
iqet	feget
uvih	guhiz
uwux	cusij
oquv	peqeg
ucuj	qavew
uhew	hejic
ihiq	qafij
afuj	fixej
oxok	cacij
egej	jufeh
iqaj	fowiv
ukuh	joqiw
uduj	qosid
uyuw	qupof
okiw	gijek
uluy	gabiy
egub	biquc
omuq	qijaq
oqig	qoyih
ixij	ditij
iceh	jigej
ojiy	guqip
ujoq	gufek
oqug	jikeq
uriw	qeyaf
uyiw	guhuy
udik	fucuj
iquq	gewih
ufof	qupef
iriw	qaviw
iwih	fepoj
uxej	geqog
uruw	qefib
uzup	gogib
iyaj	leheq
iyuf	cahiy
uxuj	cijej

aqej	hisoq
esih	giqic
uxuk	cuwuq
ujih	cixuq
oqiq	quhuq
eyik	qixew
iruw	gihin
uyoq	qaqep
igay	gayij
ogiw	gikif
owoq	bibiw
exuw	hegug
usiy	fihuw
uxij	qiyag
ihih	nufiw

Note on Replication

Data Collection with CurmElo

Data Collection can be accomplished in two ways: using the custom CurmElo software and using the CurmElo for Qualtrics script. The software for both of these methods is in the `curmelo-software subdirectory` of the main replication package directory.

0.0.1 Data Collection using the custom CurmElo software

Set up a Firebase Realtime Database instance using the instructions here: `https://firebase.google.com/docs/database/`. Then set `fbase_URL` to the URL of your firebase instance, replacing `INSERT_FIREBASE_URL_HERE`. Set a unique `run_name` each time you are ranking a new corpus. Set filename to be the filename of your corpus, which should be a text file containing a separate object from the set you want to rank on each line. Currently, it is a text file (`example_objects_to_rank.txt`) containing some three-letter test identifiers. Modify the html in the h2h function to customize the welcome message and question asked at each comparison. With a bit of work, you can make it display images, play sounds or videos, or show interactive animations – the basic structure of the system shouldn't change. Modify `num_q_per_worker` in h2h to change the number of comparisons each rater is asked to make. You can deploy this app using python's built-in test HTTP server or using gunicorn using `run_curmelo.sh` and `run_curmelo_gunicorn.sh`. You can also use other web servers using WSGI or similar. Once you've set up CurmElo, send your raters to `YOUR_DEPLOYMENT_URL/headtohead`.

0.0.2 Data Collection with CurmElo for Qualtrics

We created a python script that can generate a list of pairwise comparison questions that can be imported into Qualtrics and used with the Qualtrics platform. The file `curmelo_for_qualtrics.py` takes a CSV file with all alternatives in one column with no headers as input and outputs a file of questions that can be imported into qualtrics. Inside the script the user can set the text of the comparison question. It is not recommended to use CurmElo for Qualtrics when the set of alternatives is large as there are some randomization settings within Qualtrics that seem to need to be done for each individual question by hand.

0.0.3 Identifier Generation and Pruning

Scripts to generate and prune the four and five letter identifiers are in the `curmelo-identifier-generation-and-pruning` subdirectory of the main replication package directory. These `generate_and_prune_fiveletter_idens.py` and `generate_and_prune_fourletter_idens.py` scripts do what you'd expect them to.

All software and data files referenced in the sections below are in the `curmelo-data-and-analysis` subdirectory of the main replication package directory.

0.0.4 Creating an Elo ranking

Use the `elo_calc_fbase.py` script to produce a ranking. The `sanity_check_results_amt.py` script can be used to ensure that their are no inconsistencies between the worker log and the CurmElo data if you use Amazon Mechicanical Turk to recruit your raters.

JSON files of our own collected data are also available in the `curmelo-data-and-analysis` subdirectory under the names `4_names_4l_400_v2.json` and `5_names_1000_random_400_workers.json`. The rankings produced by these data files are stored in `4_names_4l_400_v2_mean_1000_kfac_20_rpa_400_elo.json` and `5_names_1000_random_400_workers_mean_1000_kfac_20_rpa_400_elo.json` respectively.

0.0.5 Monotonicity breaks (polarization) and transitivity breaks

The file `polarization_final.py` is a direct replication file for recreating the analysis related to Polarization, Transitivity Breaks and the Intra Tranche Win Rate. The `file polarization_functions.py` is designed to be used as an import file and contains documentation for all the functions used in `polarization_final.py`. To reproduce the bar plots used in the paper use the file `bar_plot.py`. We also include the file `elo_analysis.py`, which runs some normality tests on the Elo distributions as well as generates some counterfactual distributions. It turned out that this work was not needed but for completeness sake we included it here.

0.0.6 Phonological analysis

The file `ling_analysis.py` is a direct replication file for recreating the phonological analysis used in this paper. This script generates dummy variables for the various phonological patterns we test using pandas string manipulation. These are then used in the regressions described in the paper.
